# Back to the Future of qEEG: Lifespan Normative Modeling of Spectral Ratios and Functional Indices with Potential Applications to Therapeutic Monitoring

**DOI:** 10.1007/s10548-026-01240-4

**Published:** 2026-07-31

**Authors:** J. Bosch-Bayard, J. Guerrero-Sauzameda, R. I. Bosch-Bayard, R. Pérez-Elvira, A. Bosch-Castro, J. Sánchez-Rodríguez, A. Flores, K. Flores, E Resendiz-Flores, A. Ferrando, P. Ferrando, L. Galán-García, F. Mushtaq, P. Valdes-Sosa, G. A. Chiarenza, R. J. Biscay, L. Morales-Chacón

**Affiliations:** 1 Faculty of Psychology, Carl von Ossietzky Universität Oldenburg, Oldenburg, Germany; 2 Centro Integrador del Movimiento, Mente y Conducta (CIMMCO), Querétaro, México; 3 State University of Zanzibar, Zanzibar, Tanzania; 4 Department of Psychobiology, Faculty of Psychology, Pontifical University of Salamanca, Salamanca, Spain; 5 Neuropsychophysiology Laboratory, NEPSA Rehabilitación Neurológica, Salamanca, Spain; 6 Neuropulse, Neurocare Center, Encarnación, Paraguay; 7 Cuban Neuroscience Center, Havana, Cuba; 8 University of Leeds, Leeds, UK; 9 MOE Key Lab for Neuroinformation, School of Life Science and Technology, The Clinical Hospital of Chengdu Brain Science Institute, University of Electronic Science and Technology of China, Chengdu, China; 10 Centro Internazionale Disturbi di Apprendimento, CIDAAI, Attenzione, Iperattività, Milano, Italy; 11 Centro de Investigaciones en Matemática (CIMAT), Guanajuato, Mexico; 12 Universidad Internacional de La Rioja, Logroño, Spain; 13 Universidad de Ciencias Médicas de La Habana, Havana, Cuba

**Keywords:** Quantitative EEG, Normative models, GAMLSS, Spectral ratios (theta/beta, arousal), Electrode-level spectral normalization, Therapeutic monitoring

## Abstract

Quantitative EEG (qEEG) provides objective, millisecond-resolution measures of brain dynamics. Despite decades of methodological advances, clinically relevant derived indices—spectral power ratios, cognitive-emotional state markers, and physiological parameters—are typically reported as raw values without the normative context required for individualized clinical inference. To develop the first systematic age-dependent normative models for this family of derived qEEG indices using a multinational database, enabling probabilistic Z-score interpretation at the individual level with potential applications in objective therapeutic monitoring. Normative modeling was applied to the HarMNqEEG database (*n* = 1,564 neurologically healthy participants, ages 5–97, 9 countries, eyes-closed resting state). Electrode-level Spectral Normalization (ESN) removed inter-individual and inter-device amplitude variability while preserving the neurophysiological interpretability of each index. Age-dependent normative trajectories were estimated using Generalized Additive Models for Location, Scale, and Shape (GAMLSS) with P-splines on log(age), allowing conditional mean and variance to vary non-linearly across the lifespan. GAMLSS modeling revealed significant non-linear age-dependent trajectories for all indices. Slow-wave-dominated ratios showed steep decreases from childhood to early adulthood, consistent with cortical maturation; alpha-dominated indices increased during adolescence before stabilizing. ESN normalization yielded well-calibrated normative residuals across the full age range for all indices, except Valence. For the Valence index, a quantile regression model is provided as the recommended normative reference due to its spike-and-slab marginal distribution. These normative models provide a principled, age-adjusted probabilistic framework for individual-level qEEG interpretation, based on eyes closed resting state recordings, laying the methodological groundwork for future clinical validation in diagnostic and therapeutic monitoring applications. The ESN strategy requires no knowledge of recording equipment, ensuring broad applicability across clinical and research settings.

**Supplementary Information:**

The online version contains supplementary material available at https://doi.org/10.1007/s10548-026-01240-4.

## Introduction

The electroencephalogram (EEG) occupies a singular position among clinical neurophysiological tools. Unlike functional magnetic resonance imaging (fMRI) or positron emission tomography (PET), which measure hemodynamic and metabolic correlates of neural activity with temporal resolution on the order of seconds, EEG directly records the electrical potentials generated by the postsynaptic currents of cortical neuronal populations with millisecond temporal resolution (Michel et al. 2019; Rajkumar et al. 2021). This direct access to neuronal dynamics, combined with its relatively low cost, portability, and bedside applicability, preserves the EEG’s irreplaceable position in clinical neurology, cognitive neuroscience, and neurorehabilitation.

**Fig. 1:**
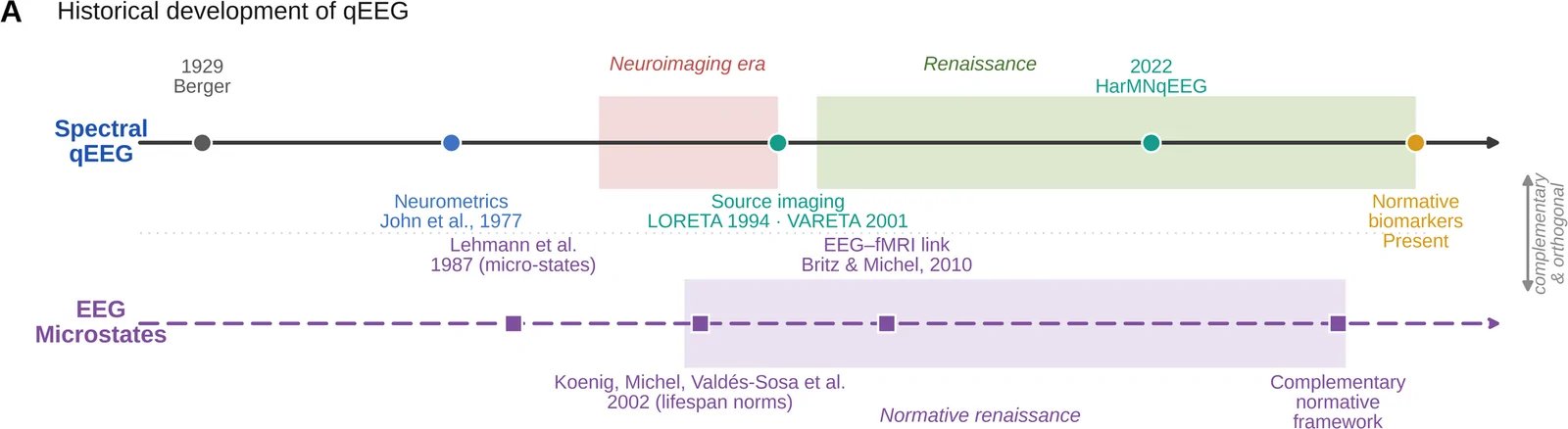
Back to the Future of quantitative EEG: historical development, conceptual evolution, and clinical translation pipeline. Historical timeline. Key milestones in EEG analysis from Berger’s first recording (1929) to the present. The red band marks the relative decline of qEEG during the neuroimaging era (1990s), when MRI, fMRI, and PET shifted clinical and scientific attention toward hemodynamic modalities. The green band marks the ongoing renaissance, driven by three convergent lines of methodological progress: the development of distributed electromagnetic source imaging methods — including LORETA (Pascual-Marqui 2002) and qEEG VARETA (Bosch-Bayard et al. 2001), which extended quantitative EEG analysis to the cortical source level; the formalization of EEG microstate analysis as a normative framework for global spatiotemporal field dynamics (Lehmann et al. 1987; Koenig et al. 2002; Michel and Koenig 2018); and the availability of large multinational normative databases such as HarMNqEEG (Li et al. 2022)

The history of EEG analysis has followed a pendular trajectory, summarized in Fig. 1A. Beginning in the 1970s and 1980s, the development of qEEG introduced a fundamental conceptual shift: EEG features could be extracted mathematically, characterized statistically, and interpreted against population norms, with neurometrics (John 1977; John et al. 1988) and age-referenced spectral databases (Matoŭek and Petersen 1973) providing the conceptual and empirical foundations. The 1990s saw a relative decline in qEEG’s clinical prominence as structural and functional neuroimaging captured the field’s attention (Turner 2021), but from the early 2000s a sustained renaissance has consolidated, driven by advances in distributed electromagnetic source imaging (Bosch-Bayard et al. 2001; Pascual-Marqui 2002) and the availability of large-scale multinational normative databases (Li et al. 2022; Valdes-Sosa et al. 2021; Bosch-Bayard et al. 2020).

Alongside these spectral and source imaging advances, a complementary tradition developed around EEG microstates—quasi-stable topographic configurations of the scalp potential field whose normative age-dependent trajectories, fMRI network correlates, and clinical sensitivity have been extensively characterized (Lehmann et al. 1987; Koenig et al. 2002; Michel and Koenig 2018). The present work occupies a parallel and complementary niche: while microstate analysis characterizes the spatiotemporal architecture of brain electric fields, the spectral ratio and functional indices addressed here capture the frequency-domain composition of those same fields, providing candidate biomarkers with direct electrode-level interpretability (see Sect.  4.4).

Despite this methodological progress, routine clinical practice remains predominantly qualitative. EEG reports continue to rely primarily on expert visual descriptions, and, at best, on raw topographic maps of spectral power. The quantitative potential of normative maps, Z-scores, and derived functional indices is frequently underused. As a consequence, valuable information regarding how an individual’s brain activity deviates from an age-typical population rarely enters formal clinical decision-making (Ko et al. 2021).

A critical limitation of current clinical workflows is the reliance on raw pre and post–treatment comparisons. Without reference to a normative framework, any observed change in an EEG index remains ambiguous: it is impossible to determine whether the observed shift represents movement toward neurotypical function, persistence within an abnormal range, or merely state-dependent fluctuation. Normative Z-score frameworks directly address this limitation by anchoring individual measurements to age-dependent population norms, enabling the clinician to quantify both the magnitude of initial deviation and the direction and significance of longitudinal change.

This longitudinal sensitivity defines the clinical application of therapeutic monitoring, which constitutes the second motivation of the present work alongside its normative aims. When a patient’s qEEG profile is expressed as a deviation from age-matched population norms, repeated assessments before and after an intervention yield a trajectory in Z-score space that is interpretable without reference to raw spectral units or device-specific calibration. Crucially, this individual-level monitoring use case is distinct from — and complementary to — supervised classification approaches that assign a categorical diagnostic label from a predefined set: a normative Z-score framework quantifies the degree and direction of individual deviation without presupposing a diagnostic category, making it applicable to heterogeneous populations and to longitudinal contexts where group identity is unknown, mixed, or evolving across the course of treatment. The proof-of-concept clinical validation presented in Sect.  3.4 directly instantiates this therapeutic monitoring use case.

An additional gap concerns the limited exploitation of multiple physiological states within qEEG protocols. Parameters assessed during eyes-open recording—such as alpha rhythm reactivity to eye opening—as well as responses to activation procedures (hyperventilation, photic stimulation) and EEG dynamics under cognitive load, provide complementary windows onto neurophysiological integrity absent from resting eyes-closed recordings alone. The individual alpha frequency (IAF) and its age-related developmental trajectory constitute particularly sensitive markers of neurophysiological maturation and thalamocortical integrity (Klimesch 1999; Pfurtscheller and Lopes da Silva 1999), yet they are seldom quantified within normative frameworks in routine clinical reports.

This limitation is clinically relevant because some electrophysiological abnormalities may remain subtle at rest but become evident during cognitive activation. In clinical practice, Swingle has explicitly recommended complementing eyes-closed and eyes-open conditions with a brief cognitive challenge such as reading or counting backwards, noting that certain patterns emerge only when the patient is cognitively challenged (Swingle 2015). From a physiological perspective, the transition from eyes-closed to eyes-open already constitutes a shift toward higher EEG arousal (Barry and De Blasio 2017; Barry et al. 2007). Beyond this basic manipulation, mental arithmetic and serial subtraction paradigms offer a practical and well-supported way to induce graded cognitive load without additional materials (Fairclough et al. 2024), while backward digit span provides a brief probe of working-memory manipulation (Onton et al. 2005). Although normative lifespan references for these activation conditions remain largely unavailable, their integration into future normative protocols would substantially enrich the clinical interpretability of qEEG-derived biomarkers.

Although several normative qEEG databases have been developed for spectral power and related Z-score inference, and harmonized multinational norms are now available for cross-spectral EEG measures, the literature remains comparatively sparse regarding clinically standardized normative references for derived frequency ratios and composite functional metrics. Indices such as theta/beta, delta/beta, frontal alpha asymmetry-related measures, arousal-related EEG markers, and engagement metrics have been widely studied, but mostly as research variables, group discriminators, or task-sensitive markers rather than as integrated age-referenced biomarkers for individualized clinical inference.

The present study aims to fill this gap by providing the first systematic normative models for this family of indices derived from the HarMNqEEG project database (Li et al. 2022), comprising 1,564 neurologically healthy subjects from 9 countries spanning the full human lifespan (ages 5 to 97). We derive age-dependent normative trajectories and individualized Z-scores from Eyes Closed resting state recordings and define a unified normative framework with electrode-level spectral normalization (ESN) to ensure applicability across the full range of clinical recording contexts.

## Materials and Methods

### The HarMNqEEG Multinational Database

The normative data used in this study were derived from the Harmonized-Multinational qEEG Norms (HarMNqEEG) project, an international collaborative initiative coordinated by the Global Brain Consortium (GBC) (Li et al. 2022). The database comprises resting-state EEG recordings from 1,564 neurologically healthy subjects, spanning the full human lifespan from 5 to 97 years of age, collected across 9 countries: Cuba, China, Malaysia, Russia, Germany, Switzerland, Canada, Colombia, and the United States. Data was obtained from 14 independent studies conducted with 12 different EEG acquisition systems, reflecting the diversity of equipment and protocols encountered in international multicenter research. The complete dataset — including subject identifiers, recording metadata, and cross-spectral matrices — is publicly available on Synapse (ID: syn26712979; https://www.synapse.org/Synapse:syn26712979/wiki/615205). The 1,564 subjects analyzed in the present study correspond exactly to the full published cohort of Li et al. (2022); no additional subject selection was performed beyond the quality criteria documented in that publication.

#### Inclusion and Exclusion Criteria

Participants were selected according to strict clinical criteria designed to constitute a representative healthy population rather than a highly selected “super-normal” sample. Exclusion criteria included: history of neurological or psychiatric disorders; current or recent use of psychoactive medications or substances with known effects on the central nervous system; significant head trauma or loss of consciousness; systemic diseases with documented neurological impact (e.g., uncontrolled diabetes mellitus, severe arterial hypertension); and abnormalities identified on neurological examination. Where available, structural MRI and cognitive screening results were also used to confirm neurological health.

### Data Preprocessing

A central challenge in aggregating EEG data across multiple recording sites and device types is the presence of systematic inter-site variability—commonly referred to as “batch effects”—that can introduce spurious differences unrelated to neurophysiology. The pipeline used here addressed this through the following procedure:

- **Artifact Rejection**: Artifact rejection procedures varied across contributing sites: in a subset of recordings, artifact-free epochs were selected through visual inspection by expert neurophysiologists; in others, automated quality-control algorithms were applied. In all cases, a minimum of 60 s of stationary EEG activity was required per participant. We acknowledge that the use of automated rejection in part of the database introduces heterogeneity in preprocessing quality. As demonstrated by Hu et al. (2025a, b), automated pipelines without expert supervision are susceptible to both insufficient preprocessing — retaining residual artifacts — and excessive preprocessing — discarding genuine brain signal — each of which produces systematic biases in downstream spectral and connectivity estimates. This constitutes a recognized limitation of the HarMNqEEG database in its current form. The PaLOSi index proposed by Hu et al. (2025a, b) provides a promising quality-control metric that could be applied uniformly across sites in future iterations of the normative pipeline to identify and flag recordings in the insufficient or excessive preprocessing range.
- **Spectral Estimation**: Cross-spectral matrices were computed for the standard 19-electrode array of the International 10–20 system using the Fast Fourier Transform (FFT) with a frequency resolution of 0.39 Hz.
- **Electrode-level Spectral Normalization (ESN)**: For each subject i and electrode ch, the Electrode-level Spectral Normalization factor is defined as ESN(ch, i) = mean_f[S(ch, f, i)] for f ∈ [0.39, 19.11] Hz, where S(ch, f, i) is the spectral power density at electrode ch and frequency f, and the average is taken over all frequency bins used in index computation. Each spectral value is then normalized by division: S_norm(ch, f, i) = S(ch, f, i) / ESN(ch, i) (5). On a log scale, this is an additive correction — equivalent to a multiplicative factor on a linear scale. The ESN simultaneously accounts for individual-level variability in scalp and skull conductivity and other multiplicative amplitude offsets that are flat across the analysis band. This universality is a key advantage over device-covariate approaches: ESN normalization is always applicable, including when the acquisition system is unknown, which is common in clinical practice. The normalization range [0.39, 19.11 Hz] was chosen to coincide with the spectral support of all indices computed in this study (TBR, TAR, DBR, Arousal, and Valence), ensuring that the ESN factor reflects exclusively the frequency content relevant to the metrics it adjusts. It also coincides with the frequency range used in Bosch-Bayard et al. (2001) for the EEG spectral norms. The upper limit was set deliberately below 20 Hz to avoid the region where device-specific high-frequency roll-off differences become non-negligible in clinical EEG amplifiers. All certified amplifiers in the IEC 60601-2-26 standard exhibit flat frequency response (± 3 dB) across the 0.5–70 Hz range; above ~ 20 Hz, however, inter-device variability in filter implementation may introduce spectral shape differences that would distort the normalization factor if included. ESN as implemented here is therefore specifically appropriate for sub-20 Hz spectral ratio indices and should not be assumed to generalize to higher-frequency biomarkers such as gamma-band or high-frequency oscillation metrics.
- **Residual variability**: after ESN normalization, residual inter-site amplitude variability is absorbed within the normative dispersion term of the GAMLSS model, since the per-electrode ESN correction removes both individual-level and external-level multiplicative scale differences before index computation.

**Fig. 2:**
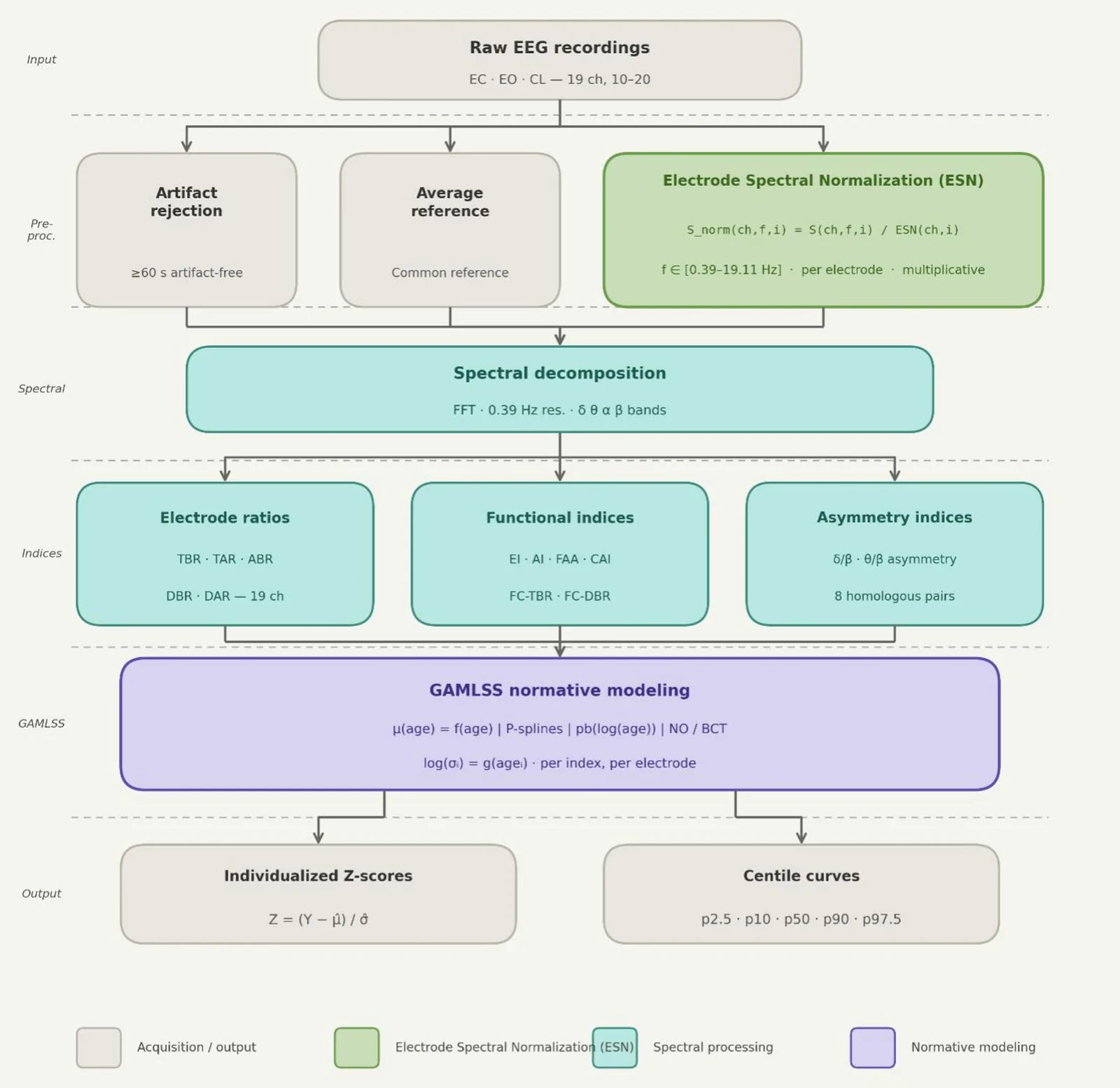
Computational pipeline for normative qEEG index modeling. Acquisition (gray): Resting-state EEG recordings from the HarMNqEEG database (*n* = 1,564; ages 5–97 years; 9 countries) under eyes-closed (EC), eyes-open (EO), and cognitive load (CL) conditions, 19-electrode 10–20 system. Preprocessing (gray): Three sequential steps — (1) artifact rejection retaining a minimum of 60 s of stationary, artifact-free signal; (2) re-referencing to the common average reference; and (3) Electrode-level Spectral Normalization (ESN), in which each electrode’s spectral values are divided by their mean power across the fixed frequency range [0.39–19.11 Hz]. This operation is multiplicative on the power scale and additive on the log scale, simultaneously removing individual-level variability in scalp and skull conductivity and other external-level variability in electrode impedance. Spectral decomposition (teal): Cross-spectral matrices computed via FFT at 0.39 Hz frequency resolution; power estimated in four canonical bands: delta (δ), theta (θ), alpha (α), and beta (β). Index computation (teal): Three families of derived indices — electrode-level spectral ratios (TBR, TAR, ABR, DBR, DAR; one normative model per electrode, 19 total); global functional state indices (EI, AI, Valence, CAI, FC-TBR, FC-DBR); and hemispheric asymmetry indices (δ/β and θ/β ratio asymmetries across 8 homologous electrode pairs). GAMLSS normative modeling (purple): A Generalized Additive Model for Location (µ), Scale (σ), and Shape (GAMLSS) was fitted per index and per electrode in R using P-spline smoothers on log(age) [pb(log(age))]; conditional distribution family selected adaptively — Normal (NO) for kurtosis ≤ 5, Box–Cox t (BCT) otherwise. Outputs (gray): Individualized Z-scores and smooth centile curves (p2.5, p10, p50, p90, p97.5) for each index and electrode across the full lifespan (ages 5–97 years)

The complete computational pipeline proposed in this work is summarized in Fig. 2. It includes Eyes Open (EO) and Cognitive Load (CL), although at present, normative databases are available only for the Eyes Closed (EC) condition. It is our purpose to standardize some Cognitive Load tasks in the future (as proposed in Sect.  4.5.1) and gather databases recorded under these parameters to calculate norms for these physiological tasks, which can provide useful information for the clinical use of qEEG.

### Calculation of Quantitative Indices

For each participant in the database, 18 quantitative indices were calculated from the spectral power densities obtained after preprocessing. Four canonical frequency bands were defined: Delta (δ: 1.5–3.5 Hz), Theta (θ: 3.9–7.5 Hz), Alpha (α: 7.9–12.5 Hz), and Beta (β: 12.9–19.14 Hz). Spectral power at a given electrode ch and band is denoted P_band(ch). Where an index is computed over a set of electrodes, the mean power across that set is used. All ratio-based indices are computed on log-transformed band powers, so that a ratio becomes a difference of logarithms: ln(P₁) − ln(P₂) ≡ ln(P₁/P₂). This log-ratio formulation is symmetric, improves distributional properties, and is standard in qEEG normative modeling (Bosch-Bayard et al. 2001; Arns et al. 2013).

All indices reported in the main text use the narrowband beta definition (β: 12.9–19.14 Hz). The High-Beta sub-band (β_high: ~15–30 Hz) used in Supplementary Figs. 5–6 and 8 is an exploratory supplement and does not enter the normative models.

#### Electrode-Level Spectral Ratios

The following three ratios are computed separately for each of the 19 electrodes of the 10–20 system, yielding 19 values per ratio. Each electrode–ratio combination receives its own age-dependent normative model.

- **Theta/Beta Ratio (TBR**,** per electrode)**: TBR(ch) = ln[θ(ch)] − ln[β(ch)]. Widely used as a marker of attentional regulation and cortical arousal (Arns et al. 2013; Ogrim et al. 2012).
- **Theta/Alpha Ratio (TAR**,** per electrode)**: TAR(ch) = ln[θ(ch)] − ln[α(ch)]. Reflects the relative dominance of theta over alpha; sensitive to vigilance level and cognitive fatigue (Klimesch 1999).
- **Alpha/Beta Ratio (ABR**,** per electrode)**: ABR(ch) = ln[α(ch)] − ln[β(ch)]. Indexes the balance between idling alpha states and active beta processing; elevated values are associated with reduced cortical arousal and drowsiness.
- **Delta/Beta Ratio (DBR**,** per electrode)**: DBR(ch) = ln[δ(ch)] − ln[β(ch)]. Captures the balance between slow delta oscillations and fast beta activity; elevated DBR has been associated with cortical hypoactivation and is sensitive to disorders of consciousness and severe attentional dysregulation (Thatcher et al. 1989).
- **Delta/Alpha Ratio (DAR**,** per electrode)**: DAR(ch) = ln[δ(ch)] − ln[α(ch)]. Widely used as an indicator of cerebral ischemia, diffuse encephalopathy, and cognitive impairment, elevated DAR reflects pathological slow-wave dominance (Claassen et al. 2024).
- **Delta/Theta Ratio (DTR**,** per electrode)**: DTR(ch) = ln[δ(ch)] − ln[θ(ch)]. Reflects the relative contribution of infra-slow delta versus theta activity; sensitive to depth of sleep and severe cortical depression, and useful for distinguishing delta-dominant from theta-dominant slow-wave profiles.

#### Global Functional State Indices

These four indices characterize broad functional brain states and are computed from spatially averaged power across all 19 electrodes (denoted β̅, α̅, θ̅, δ̅), except where noted.

- **Engagement Index (EI)**: EI = β̅ / (α̅ + θ̅), following Pope et al. 1995). Computed over all 19 electrodes. Captures the relative suppression of slow-wave activity during sustained cognitive engagement.
- **Arousal Index (AI)**: AI = β_F̅ / (δ_F̅ + θ_F̅), where the overbar denotes the mean over the frontal cluster F3, F4. Quantifies the balance between frontal fast and slow oscillations as a measure of CNS activation level (Bonnet and Arand 2007).
- **Valence Index**: Valence = α(F3)/β(F3) − α(F4)/β(F4), computed from the alpha power at electrodes F3 and F4 individually. Operationalizes the frontal EEG asymmetry framework linking hemispheric alpha lateralization to emotional valence (Allen et al. 2018; Davidson 1998).
- **Cognitive-Affective Index (CAI)**: CAI = FC-TBR × Valence, where FC-TBR is the frontocentral theta/beta ratio defined in Sect.  2.3.3, and Valence is defined above. This compound index captures the simultaneous contribution of frontocentral cognitive load and hemispheric emotional lateralization.

#### Frontocentral Regional Ratios

These two indices restrict the spectral ratio computation to the frontocentral cluster comprising electrodes F3, F4, and Cz, yielding a single value per index, the mean ratio across those three electrodes.

- **FrontoCentral Theta/Beta (FC-TBR)**: FC-TBR = ln[θ_FC̅] − ln[β_FC̅], where the overbar denotes the mean over F3, F4, Cz. The regional counterpart of the per-electrode TBR, focusing on the frontocentral region relevant to executive control and attentional regulation (Arns et al. 2013).
- **FrontoCentral Delta/Beta (FC-DBR)**: FC-DBR = ln[δ_FC̅] − ln[β_FC̅], computed over the same cluster F3, F4, Cz. Captures the balance between frontocentral delta and beta activity.

#### Hemispheric Asymmetry Indices

Two families of asymmetry indices are computed, both using the log-difference formulation:

  

$$\begin{aligned}Asymmetry\,=&\,ln\left[ {M\left( {left{\text{ }}electrode} \right)} \right]\, \\&- \,ln\left[ {M\left( {right{\text{ }}electrode} \right)} \right]{\rm (A)}\end{aligned}$$

where M is either absolute band power or a spectral ratio. A positive value indicates left-hemisphere dominance; a negative value indicates right-hemisphere dominance.

#### a. Asymmetry Index (per band, per homologous pair)

The Asymmetry Index is computed as the log-difference of absolute band power between homologous electrode pairs, separately for each of the four canonical frequency bands:

  

$$\begin{aligned}AI\left( {band,{\text{ }}pair} \right)\,=&\,ln\left[ {P\_band\left( {left} \right)} \right]\, \\&- \,ln\left[ {P\_band\left( {right} \right)} \right]{\rm (B)}\end{aligned}$$

This yields a matrix of values indexed by band (δ, θ, α, β) and homologous pair. The pairs used are: Fp1/Fp2, F3/F4, F7/F8, C3/C4, T3/T4, T5/T6, P3/P4, and O1/O2. Each band–pair combination receives its own age-dependent normative model.

#### b. Ratio-Based Asymmetry Indices

Eight indices combine spectral ratios with hemispheric asymmetry, computed at four clinically selected pairs of homologous electrodes using two ratios (δ/β and θ/β):

  

$$Asym\left( {ratio,{\text{ }}pair} \right)\,=\,ln\left[ {ratio\left( {left} \right)} \right]\, - \,ln\left[ {ratio\left( {right} \right)} \right]{\rm (C)}$$

The eight indices are (age-dependent normative curves shown in Supplementary Fig. 7):

- **δ/β Asymmetry F3 − F4**: ln[δ(F3)/β(F3)] − ln[δ(F4)/β(F4)].
- **θ/β Asymmetry F3 − F4**: ln[θ(F3)/β(F3)] − ln[θ(F4)/β(F4)].
- **δ/β Asymmetry F7 − F8**: ln[δ(F7)/β(F7)] − ln[δ(F8)/β(F8)].
- **θ/β Asymmetry F7 − F8**: ln[θ(F7)/β(F7)] − ln[θ(F8)/β(F8)].
- **δ/β Asymmetry T3 − T4**: ln[δ(T3)/β(T3)] − ln[δ(T4)/β(T4)].
- **θ/β Asymmetry T3 − T4**: ln[θ(T3)/β(T3)] − ln[θ(T4)/β(T4)].
- **δ/β Asymmetry T5 − T6**: ln[δ(T5)/β(T5)] − ln[δ(T6)/β(T6)].
- **θ/β Asymmetry T5 − T6**: ln[θ(T5)/β(T5)] − ln[θ(T6)/β(T6)].

### Normative Modeling and Z-Score Derivation

#### Distributional Transformation

The indices computed in this study are expressed as log-ratios or log-differences of band powers and therefore span negative and positive values with approximately symmetric distributions. No additional distributional transformation was applied before model fitting. This is consistent with the log-ratio formulation described in Sect.  2.3, which ensures that ratio-based indices are already on an approximately linear scale suitable for Gaussian normative modeling.

#### GAMLSS Normative Framework

Age-dependent normative trajectories were estimated using a heteroscedastic regression framework consistent with the two-parameter GAMLSS family [Rigby and Stasinopoulos 2005 ], with both the conditional mean (µ) and the log-standard deviation (log σ) modeled as smooth functions of age via penalized B-splines (P-splines).

The conditional distribution family was selected adaptively for each index using a three-criterion procedure evaluated on the raw (pre-transformation) data. Three diagnostic statistics were computed: excess kurtosis (kurt − 3), skewness, and the p-value of a Shapiro–Wilk test on a subsample of up to 2,000 observations. When at least two of the three criteria indicated non-normality (excess kurtosis > 1, |skewness| > 0.5, or Shapiro–Wilk *p* < 0.05), a non-Normal family was attempted. For indices with strictly positive values, the Box–Cox t (BCT) family was fitted on the raw data — absorbing asymmetry and heavy tails through its shape parameters ν and τ, making a preliminary log-transformation redundant — with fallback to BCPE and then Normal (NO) if BCT failed to converge (see Supplementary Table 3 for more details).

For indices with values spanning the real line (e.g., Valence), the Normal family was retained regardless of the diagnostic criteria, as empirical testing confirmed that alternative families (JSU, SHASHo) did not improve centile calibration for the spike-and-slab marginal distribution (see Supplementary Fig. 8, Valence panel) characteristic of the Valence in healthy populations. When fewer than two criteria indicated non-normality, the Normal family was used directly, with any requested pre-transformation (e.g., log for Arousal) applied before fitting.

Let Y_i_ denote the (transformed) value of a given EEG-derived index for subject i. The conditional distribution of Y_i_ is assumed to follow a parametric family:

1 $$Y_i \sim D(\mu_i,\,\sigma_i,\, \nu_i,\,\tau_i)$$

where µ_i_ is the conditional mean, σ_i_ is the conditional standard deviation, and ν_i_ and τ_i_ are optional shape parameters governing skewness and kurtosis, respectively. For most EEG indices, modeling of µ_i_ and σ_i_ was sufficient; higher-order parameters were included only when residual diagnostics indicated significant departure from distributional assumptions.

The conditional mean and dispersion were modeled as:

2 $$\mu_i =f(age_i)$$

3 $$log(\sigma_i )\,=\,g(age_i)$$

where f(·) and g(·) are smooth functions of log(age) estimated as penalized B-splines (P-splines) using the pb() smoother of the gamlss R package (Rigby and Stasinopoulos 2005). The degree of smoothing is selected automatically by minimizing a generalized Akaike information criterion (GAIC), avoiding the need to prespecify the number or location of knots. Both location and dispersion submodels are fitted jointly by alternating IRLS cycles (maximum 50 outer iterations). When residual diagnostics—specifically, worm plots and Q–Q plots of normalized quantile residuals—indicated significant departure from normality, operationalized as excess kurtosis greater than 5, the Box–Cox t (BCT) family was substituted for the Normal family to accommodate heavy-tailed distributions. For all remaining indices, the Normal (NO) conditional family was used. This adaptive family selection ensures distributional adequacy across the full set of indices, which differ substantially in tail behavior.

Given the spike-and-slab marginal distribution of the Valence index — characterized by a high concentration of values near zero and heavy tails — no tested parametric family (Normal, BCT, BCPE, JSU, SHASHo) achieved adequate calibration in the central quantile range. As an alternative, we fitted age-dependent quantile regression models for Valence using B-splines (cubic, 4 internal knots) on log-transformed age, implemented with the quantreg package (Koenker 2005) in R. This non-parametric approach achieved near-perfect calibration across all centiles (maximum |Δ| = 0.3 pp; Supplementary Fig. 13). Quantile regression Z-scores for Valence were computed as Φ⁻¹(empirical percentile), where the empirical percentile was obtained by interpolating the subject’s observed value within the estimated centile grid at their age. The GAMLSS-based Valence Z-scores are retained for consistency, but their use is restricted to extreme deviations (|Z| > 2); for the central range, the quantile regression model is recommended.

#### Electrode-level Spectral Normalization (ESN)

A key preprocessing step before normative modeling is the removal of multiplicative amplitude variability that reflects physiological and technical sources unrelated to the neurophysiological indices of interest. These sources include individual differences in scalp and skull conductivity—which scale all electrode amplitudes by a subject-specific factor—and other externally related differences in electrode impedance, which introduce electrode-specific scale offsets that vary across acquisition systems. Critically, these two sources of variability are confounded and cannot be separated without detailed knowledge of the recording hardware.

To address this, an Electrode-level Spectral Normalization factor (ESN) is computed for each subject i and electrode ch as:

4 $$\rm ESN\left( {ch,{\text{ }}i} \right){\text{ }}={\text{ }}mean\_f{\text{ }}\left[ {{\text{ }}S\left( {ch,{\text{ }}f,{\text{ }}i} \right){\text{ }}} \right],{\text{ }}f \in \left[ {0.39,{\text{ }}19.11} \right]{\text{ }}Hz$$

where S(ch, f, i) is the spectral power density at electrode ch, frequency bin f, and subject i, and the mean is taken over all frequency bins used in the computation of normative indices. The frequency range [0.39, 19.11] Hz is fixed and identical for all subjects and studies, ensuring that the ESN factor is strictly comparable across individuals and recording systems. Each spectral value is then normalized by division:

5 $$\rm S\_norm\left( {ch,{\text{ }}f,{\text{ }}i} \right)\,=\,S\left( {ch,{\text{ }}f,{\text{ }}i} \right){\text{ }}/{\text{ }}ESN\left( {ch,{\text{ }}i} \right)$$

In log-scale, this corresponds to a subtraction: ln[Sₙₒ℠ₘ] = ln[S] − ln[ESN], making the ESN correction additive on the scale on which normative models are fitted. Equivalently, on the original power scale, the correction is multiplicative. This distinction is methodologically important: multiplicative normalization preserves the relative spectral structure within each subject while removing between-subject and between other recording-conditions-related scale differences.

ESN corrects multiplicative amplitude differences per electrode within the analysis band but does not account for frequency-dependent transfer function differences across devices (e.g., differential roll-off slopes or filter characteristics). Studies combining equipment with substantially different spectral response characteristics should verify residual device effects in GAMLSS model residuals.

A fundamental advantage of ESN normalization over device-covariate approaches is that it requires no knowledge of the recording equipment. It is therefore universally applicable: it can be applied to any EEG recording, including archival data, referral recordings, and recordings from systems not represented in the normative database—contexts in which a device covariate cannot be specified. This universality ensures that the normative framework developed here can be applied in the full range of clinical and research settings encountered in practice.

Following ESN normalization, the conditional mean of the GAMLSS model contains only the age-dependent smooth term:

6 $$\rm \mu_i =f(age_i)$$

7 $$\rm log(\sigma _i)\,=\,g(age_i)$$

where f(·) and g(·) are P-spline smoothers on log(age). The individualized Z-score for subject i with index value Y_i_ is:

8 $$\rm Z_i =[Y_i-\hat{\mu}_i]/\hat{\sigma_i}$$

where µ̂_i_ and σ̂_i_ are the age-adjusted normative mean and standard deviation derived from the GAMLSS model.

#### Computation of Individualized Deviation Scores

For any individual subject i with index value Y_i_ measured at age t, the individualized Z-score under either normalization mode is interpreted as the number of age-adjusted standard deviations by which the observed measurement deviates from the normative expectation:

Under conventional probabilistic thresholds:

- |Z| > 2.0 corresponds to approximately the 2.5th or 97.5th percentile of the normative population, indicating a borderline deviation.
- |Z| > 3.0 indicates a strong deviation, expected in fewer than 0.15% of neurotypical individuals of the same age.

In addition to Z-scores, smooth age-dependent centile curves (5th, 10th, 25th, 50th, 75th, 90th, and 95th centiles) were generated for each index and electrode region, providing a reference for clinical and research use (*see Supplementary Table 2*).

#### Model Validation

The stability and predictive performance of the normative models were evaluated using several complementary procedures:

- **k-fold cross-validation**: Models were fitted on training subsets and evaluated on held-out data, with prediction error assessed separately within each age stratum.
- **Residual diagnostics**: Worm plots and Q-Q plots of normalized quantile residuals were inspected to verify distributional adequacy of the fitted models.
- **Centile calibration**: Empirical coverage of predicted centile intervals was compared against theoretical expectations across the full age range.
- **Assessment of ESN normalization and model calibration**: Assessment of ESN normalization: The reduction in inter-subject spectral amplitude variability following per-electrode ESN correction was quantified, and centile calibration was verified after normalization (*see Supplementary Fig. 10*).

Model calibration was assessed through two complementary procedures reported in Supplementary Tables 1 and 2: (1) the percentage of normative subjects with normalized quantile residuals |Z| > 1.96, computed using R’s internal quantile residual diagnostics and expected to approximate 5.0% under a well-calibrated model; and (2) empirical centile coverage, evaluated by comparing each subject’s observed value against the age-interpolated centile curve across seven thresholds (p2.5 through p97.5).

### Software and Implementation

Spectral analysis and preprocessing were implemented in MATLAB (R2015a). Normative modeling was performed in R (v4.3) using the gamlss package (Rigby and Stasinopoulos 2005; Stasinopoulos and Rigby 2007). For each index, a two-parameter GAMLSS model with P-spline smoothers on log(age) was fitted using the pb() function, with automatic smoothing parameter selection via GAIC. Inter-site and inter-individual amplitude variability was addressed through Electrode-level Spectral Normalization (ESN) applied at the electrode level before index computation (see Sect.  2.4.3) rather than as a separate preprocessing step. Topographic Z-score interpolation for electrode-level surfaces was performed using spherical spline interpolation of order 4 [Perrin et al. 1989 ].

## Results

### Distribution of EEG Spectral Indices and Transformation Assessment

The distribution of each spectral index was first examined across the full normative sample. Most indices displayed moderate skewness, typical of ratio-based measures derived from spectral power. Log-transformations were applied when appropriate to stabilize variance and improve model fit. Gaussian models for all major indices satisfactorily approximated post-transformation residual distributions.

### Age-Dependent Normative Trajectories

**Fig. 3:**
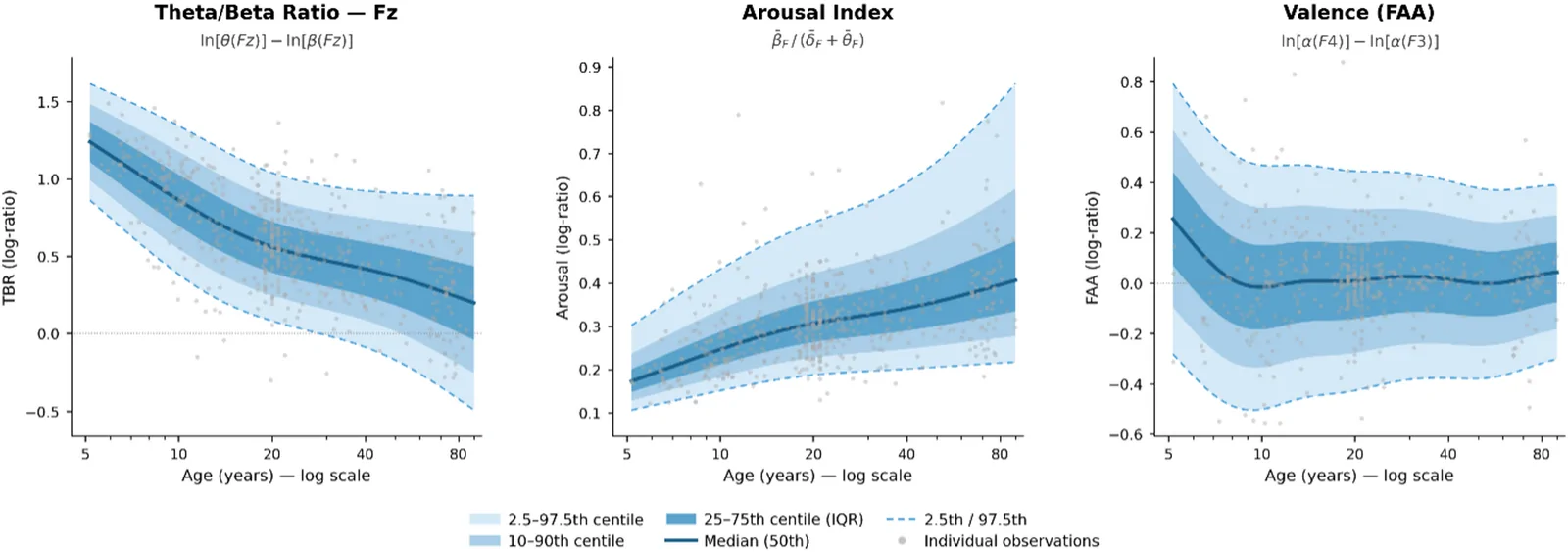
Age-dependent normative centile curves for three representative qEEG indices across the human lifespan. Each panel displays individual observations from the HarMNqEEG normative database (*n* = 1,564; ages 5–97 years; grey dots) overlaid with smooth centile curves estimated by GAMLSS with P-spline smoothers on log(age). Shaded bands correspond to the 2.5–97.5th (lightest), 10–90th, and 25–75th (darkest) centile intervals; the solid line indicates the median (50th centile); dashed lines mark the 2.5th and 97.5th centiles. The x-axis is displayed on a logarithmic scale to compress the rapid developmental changes of childhood relative to the more gradual changes of adulthood. (A) Theta/Beta Ratio at Fz: a marker of attentional regulation and cortical arousal showing a pronounced age-dependent decrease from childhood to early adulthood, consistent with progressive cortical maturation and reduction of slow oscillatory dominance (Arns et al. 2013; Ogrim et al. 2012). (B) Arousal Index: an index of CNS activation level exhibiting a gradual increase across the lifespan, with greatest inter-individual variability in older age groups (Bonnet and Arand 2007). (C) Valence Index: an index of hemispheric lateralization linked to emotional valence, showing a near-zero median trajectory across the lifespan with wide normative dispersion, reflecting the high inter-individual variability of frontal asymmetry in a healthy population (Allen et al. 2018; Davidson 1998). Note: The Valence index shows a spike-and-slab marginal distribution in healthy individuals — a high concentration of values near zero combined with heavy tails — reflecting genuine neurophysiological heterogeneity in frontal alpha/beta lateralization at rest. While the wide normative band captures this biological variability, the Normal GAMLSS family over-estimates dispersion in the central range (p25–p75); a quantile regression model with superior central calibration is provided in Supplementary Fig. 13 as the recommended reference for this index. Clinicians should exercise caution when interpreting Valence Z-scores in the intermediate range (|Z| < 2); normative interpretation is most reliable for extreme deviations (|Z| > 2)

**Fig. 4:**
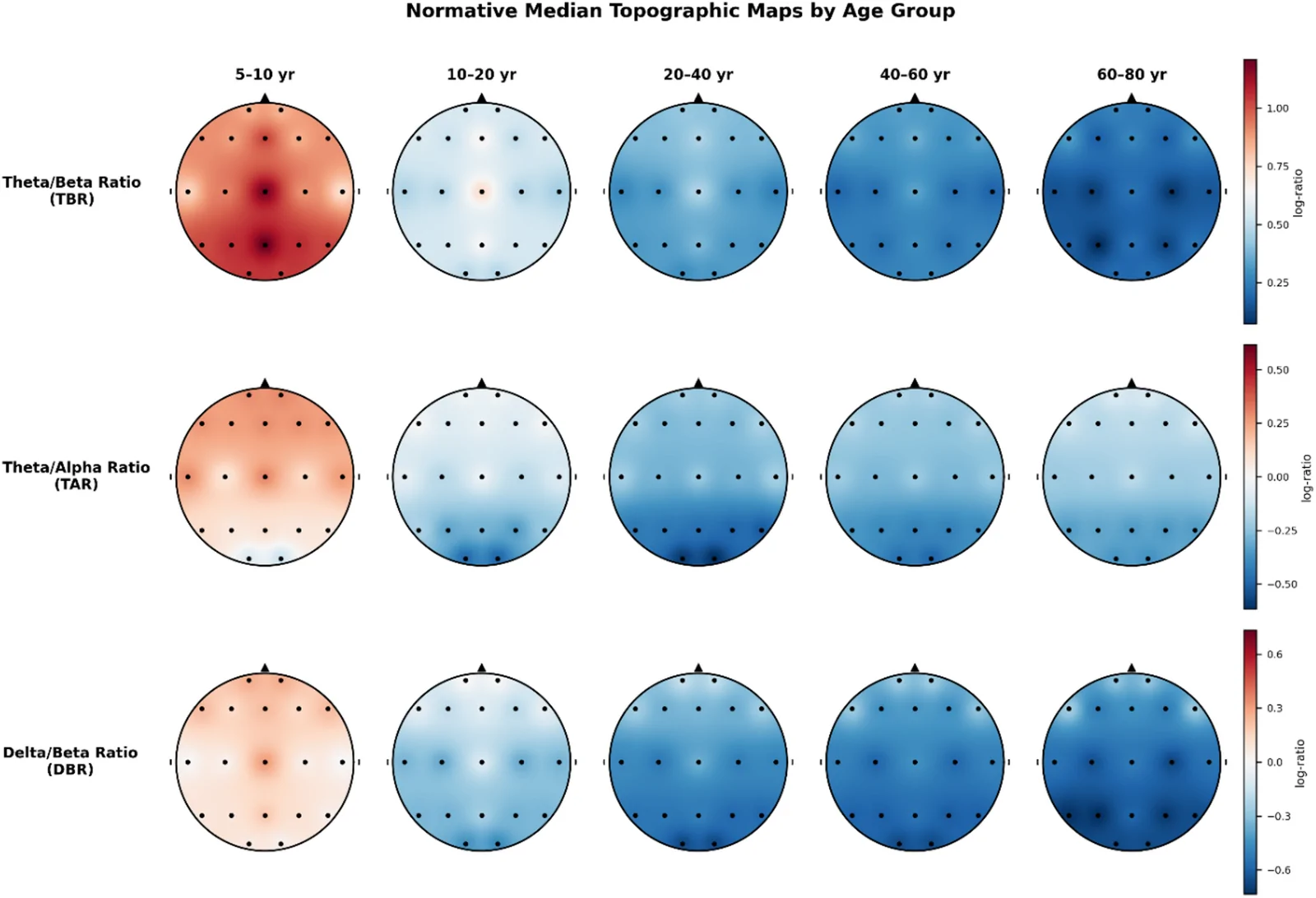
Age-dependent topographic distribution of normative median spectral ratio indices across five age groups. Each map shows the interpolated spatial distribution of the GAMLSS normative median (50th centile) across the 19 standard 10–20 electrodes, estimated at representative age-groups (5–10, 10–20, 20–40, 40–60, 60–80 years). Color scale: red = higher values, blue = lower values; shared within each row to allow direct developmental comparison. Electrode positions follow the standard azimuthal projection (nose up). **(Row 1) TBR**: pronounced fronto-central predominance in childhood, with systematic decrease across all regions into adulthood, consistent with cortical maturation and reduction of slow oscillatory dominance (Arns et al. 2013; Ogrim et al. 2012). **(Row 2) TAR**: broadly distributed slow-wave dominance in childhood transitions toward a flatter distribution in adulthood, reflecting developmental strengthening of alpha relative to theta. **(Row 3) DBR**: consistently higher values in childhood with pronounced age-dependent decrease in frontal regions, consistent with maturation of prefrontal inhibitory control. Spatial interpolation via radial basis functions (thin-plate spline). Note: absolute values differ between indices; cross-row comparisons should be interpreted within each index’s normative range

Normative trajectories estimated via GAMLSS revealed significant nonlinear developmental patterns across all computed indices (see Fig. 3 for three representative examples; complete centile trajectories for all 19 electrode-level indices are provided as Supplementary Figures). The theta/beta ratio decreased markedly from childhood to early adulthood, consistent with the maturation of attentional networks and the progressive reduction in slow oscillatory activity. The delta/beta ratio showed a similar decreasing trajectory. The Arousal Index exhibited a gradual increase across the lifespan, reflecting the progressive strengthening of fast cortical rhythms relative to slow oscillations. The Valence index showed a near-zero median trajectory with wide normative dispersion across all ages, consistent with the high inter-individual variability of frontal alpha asymmetry in healthy populations. Figure 4 displays the age-dependent topographic distribution of the normative median for TBR, TAR, and DBR across five representative age groups, illustrating the spatial evolution of slow-to-fast ratio indices from childhood to late adulthood.

Complete normative centile trajectories for all 19 electrode-level indices are provided as Supplementary Figures. Each supplementary figure displays, for a given index, the age-dependent normative mean and ± 1.96σ interval estimated by the GAMLSS model alongside individual observations from the HarMNqEEG normative database, arranged by electrode according to the standard 10–20 spatial layout.

### Normative Centile Curves and Z-score Distributions

Formal calibration diagnostics confirmed adequate model performance for all indices. In Supplementary Tables 1 and 2, we show the results for three of the most popular and used indices: Arousal, Valence, and TBR (Theta/Beta Ratio). TBR and Arousal Index models showed excellent calibration across all centile thresholds (max |Δ| < 1.4 pp and < 1.0 pp, respectively; residual rates within 0.1 pp of the nominal 5.0%). The Valence Index showed adequate global calibration (5.11% residuals outside ± 1.96σ) but systematic miscalibration in the central quantiles (p25: −10 pp; p75: +10 pp), reflecting the spike-and-slab character of the Valence distribution in healthy populations (see Supplementary Fig. 10 for the age-stratified individual distribution). This limits normative precision in the intermediate range (|Z| < 2) but does not affect the primary clinical use of this index — detection of pathological hemispheric lateralization (|Z| > 2). As described in Sect.  2.4.2, a quantile regression model was fitted as an alternative normative reference for Valence, achieving near-perfect calibration across all centiles (maximum |Δ| = 0.3 pp; Supplementary Fig. 13); this model is recommended for applications requiring normative inference in the central range. Device-stratified residual diagnostics confirmed the absence of clinically meaningful equipment-related bias across all indices (Supplementary Fig. 10; Kruskal-Wallis H(8) range: 17.85–47.07, all *p* < 0.05; η² range: 0.008–0.031; maximum mean device deviation |Z̄| < 0.75 across all indices; Supplementary Table 4).

### Potential Clinical Utility of the Normative qEEG Indices

To provide proof-of-concept evidence for the therapeutic monitoring application highlighted in the title and motivated in the Introduction, we present two complementary lines of evidence: first, a group-level illustration from an independent intervention study in older adults, showing that the normative indices discriminate clinical status and track therapeutically induced neurophysiological change; and second, a representative single-case pre–post illustration demonstrating how Z-scores anchor individual EEG changes within the population normative distribution. For the group-level illustration, we applied the normative pipeline to longitudinal data from an independent cognitive–motor intervention study (Bosch-Bayard et al. 2026). Pre- and post-intervention qEEG recordings from 19 older adults were processed through the same normative pipeline described above, and individual Z-score trajectories were computed for each index. This design directly instantiates the therapeutic monitoring use case: the same normative reference frame that characterizes a patient’s baseline deviation from age-matched norms is applied at follow-up to quantify the direction and magnitude of change, independently of absolute spectral values or recording equipment.

#### Group-level Illustration: Normative Index Sensitivity in an Independent Intervention Study

The normative qEEG indices derived from the pipeline show convergent validity with behavioural outcomes in a cognitive–motor intervention study (Bosch-Bayard et al. 2026). In an independent sample of 19 older adults (9 with Mild Cognitive Impairment [MCI], 10 cognitively healthy [HC]) who completed 30 sessions of Kinestesia—a structured cognitive–motor programme combining therapeutic videogames, rhythmic gait, and sensory stimulation—the normative Z-score framework revealed two complementary patterns of clinical sensitivity.

**Fig. 5:**
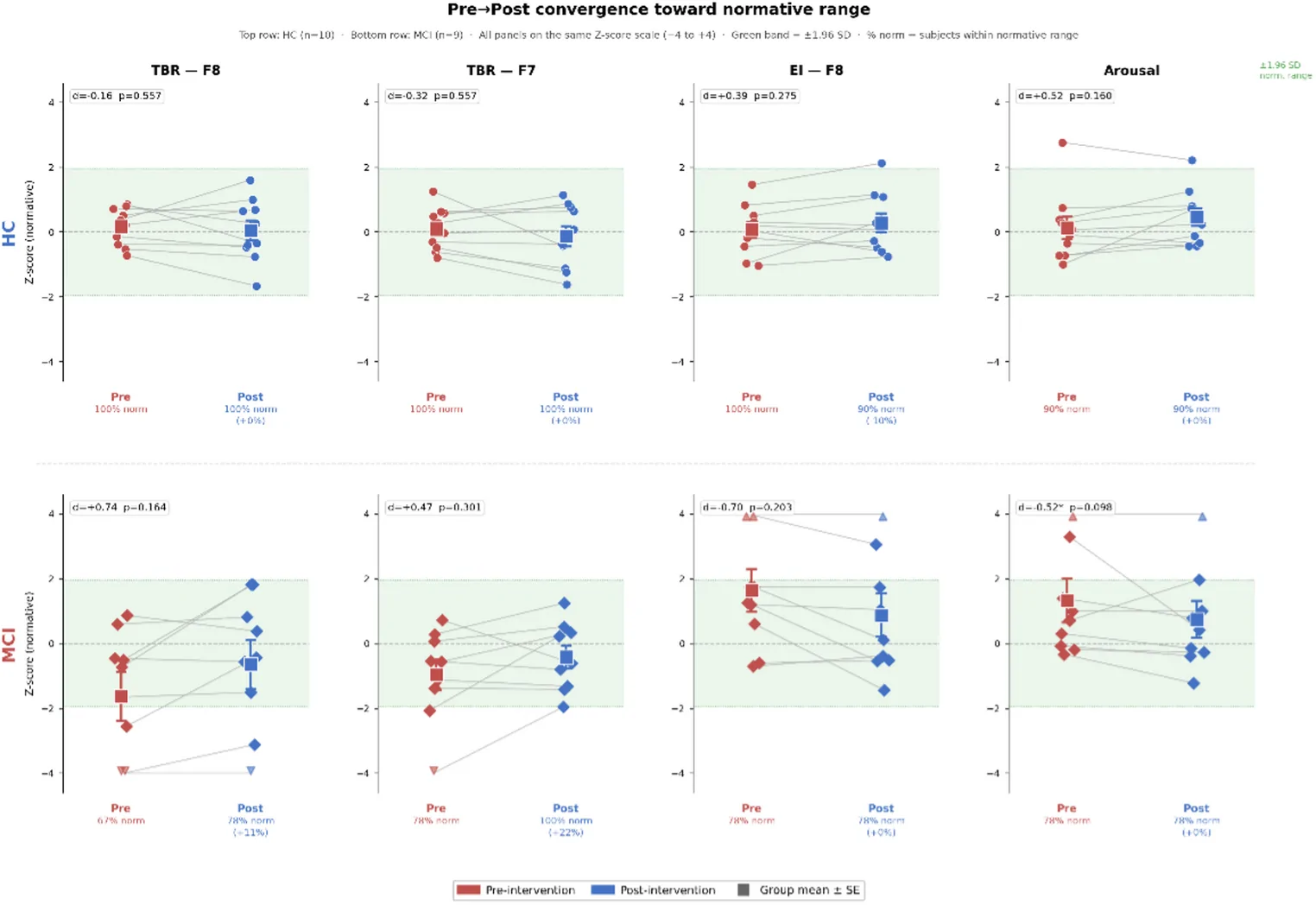
Pre–post convergence toward the normative range in an independent cohort of 19 older adults (9 MCI, 10 HC; Bosch-Bayard et al. 2026; doi:https://doi.org/10.1016/j.archger.2026.106346). Each panel displays individual Z-scores (shapes) and group mean ± SE (filled squares) before (red) and after (blue) 30 sessions of Kinestesia cognitive–motor intervention, for four representative qEEG indices (TBR-F8, TBR-F7, EI-F8, Arousal). Top row: HC group; bottom row: MCI group. The green band marks the ± 1.96 SD normative range. Percentage labels indicate the proportion of subjects within the normative band at each time point, with the delta (Δ) reflecting post–pre change. HC participants remain distributed around Z = 0 with minimal change, while MCI participants show systematically elevated pre-intervention Z-scores with greater dispersion; following the intervention, the MCI group mean shifts toward zero and dispersion decreases, indicating normalization of the neurophysiological profile. Effect sizes (Cohen’s d) and p-values from paired t-tests are reported in each panel; asterisks indicate d > 0.5

First, Fig. 5 shows the pre–post distribution of normative Z-scores for four representative indices: TBR-F8, TBR-F7, EI-F8, and Arousal (TBR-Theta/Beta Ratio, EI-Engagement Index) stratified by group. At baseline, HC participants showed Z-scores distributed tightly around zero—consistent with their membership in the age-matched normative population—while MCI participants showed systematically elevated Z-scores with greater dispersion, reflecting clinically meaningful deviation from the normative expectation (pre-intervention: 67–78% of MCI subjects within normative range vs. 90–100% of HC). Following the intervention, MCI participants showed a consistent shift of the group mean toward zero and a reduction in dispersion, with normalization rates increasing to 78–100% across indices. HC participants remained stable within the normative band. This pattern illustrates two independent properties of the normative framework: its capacity to discriminate between clinical groups at baseline, and its sensitivity to capture intervention-related neurophysiological change in the direction of normalization.

**Fig. 6:**
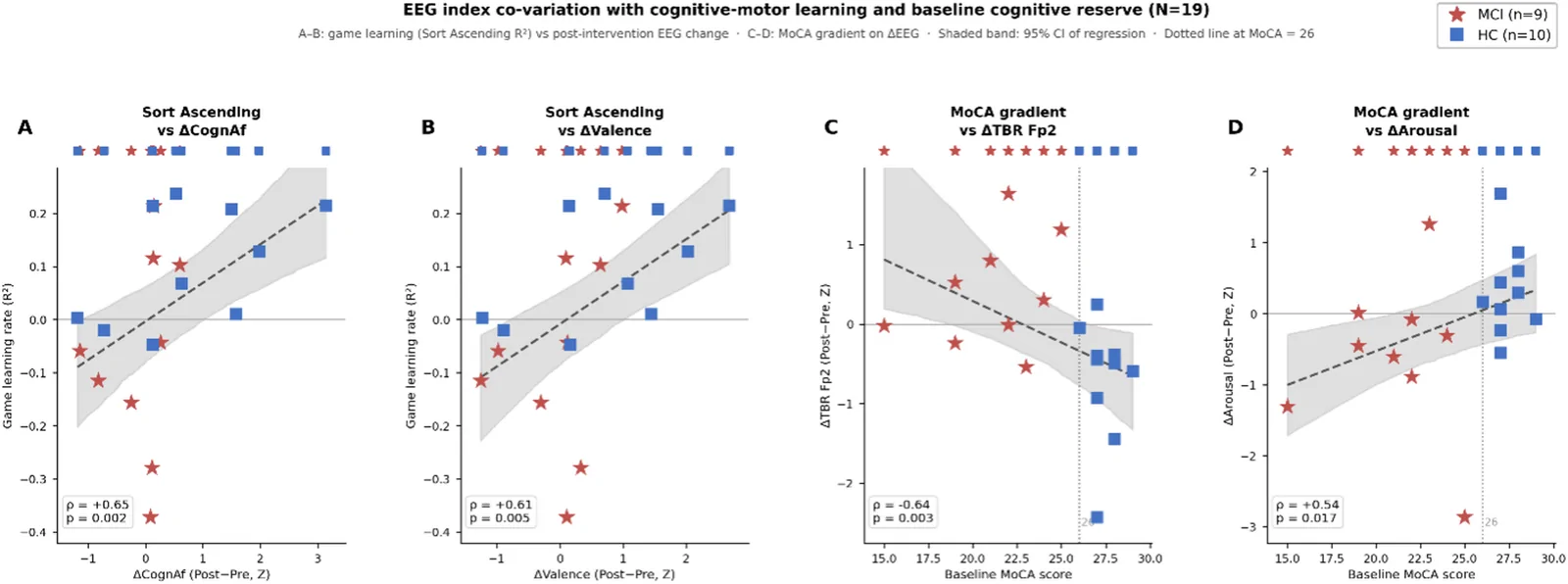
EEG index co-variation with cognitive-motor learning and baseline cognitive reserve (*N* = 19; Bosch-Bayard et al. 2026; doi:https://doi.org/10.1016/j.archger.2026.106346). Panels A–B: post-intervention change in the Cognitive-Affective composite (ΔCognAf, panel A) and Valence (ΔValence, panel B) indices (Z-score units, Post − Pre) plotted against individual game-learning rate (Sort Ascending R²). Panels C–D: MoCA gradient on post-intervention change in TBR at Fp2 (ΔTBR Fp2, panel C) and Arousal (ΔArousal, panel D). Dashed regression lines with 95% confidence bands; dotted vertical line at MoCA = 26 (MCI–HC threshold). Red stars: MCI participants; blue squares: HC participants. Spearman ρ and p-values are reported in each panel. Panels A–B demonstrate that the magnitude of EEG index normalization co-varies with cognitive-motor learning success, independently of group membership. Panels C–D demonstrate a MoCA gradient: participants with lower baseline cognitive reserve show greater intervention-related reduction in slow-to-fast ratio (TBR) and a qualitatively distinct Arousal trajectory, consistent with a larger neurophysiological distance from the norm at baseline and greater room for normalization

Second, Fig. 6 shows that the magnitude of index change co-varied with individual differences in baseline cognitive reserve and cognitive-motor learning trajectory. Post-intervention changes in the Cognitive-Affective composite (CognAf) and Valence indices co-varied significantly with individual cognitive-learning trajectories in sequential ordering tasks (ρ = +0.65 and + 0.61 respectively, *p* < 0.006; Fig. 6, panels A–B), indicating that the direction and magnitude of spectral change captured by the normative indices tracks meaningful individual differences in cognitive-motor learning rather than nonspecific EEG fluctuation. Furthermore, the TBR and Arousal indices showed a continuous gradient with baseline MoCA score: participants with lower cognitive reserve showed greater post-intervention reduction in prefrontal TBR (Fp2: ρ = −0.64, *p* = 0.003; Fig. 6, panel C) and a qualitatively distinct Arousal trajectory relative to cognitively healthy participants (ρ = +0.54, *p* = 0.017; Fig. 6, panel D). This state-dependent pattern—wherein the same intervention produces divergent index trajectories depending on the individual’s baseline neurophysiological profile—illustrates the added value of normative Z-scoring over absolute power metrics: deviations from the age-matched population mean, rather than raw spectral values, are what index the clinically relevant heterogeneity of response. All associations are exploratory and are detailed in the companion neurophysiological study (Bosch-Bayard et al. 2026).

#### Single-Case Illustration

The participant is an 8-year-old male (initials B.M., data used with institutional consent) from Mexico, presenting with a clinical diagnosis of dysexecutive syndrome—characterized by deficits in working memory, cognitive flexibility, inhibitory control, and goal-directed behavior. He received 21 sessions of transcranial direct current stimulation (tDCS) combined with cognitive-motor neurorehabilitation using the Kinestesia therapeutic video-game platform, targeting frontal executive networks. Quantitative EEG (qEEG) was recorded under standardized eyes-closed (EC), eyes-open (EO), and cognitive load (CL) conditions before and after the intervention. Broadband spectral power (absolute power, relative power, mean frequency), spectral ratios, and functional state indices were computed following the pipeline described in Sect.  2.3. For the EC condition, normative Z-scores were derived using the age-matched models developed in this study.

##### Case Description and Spectral Changes

Pre-intervention qEEG showed a global mean EEG frequency between 5.86 and 7.81 Hz — below the age-expected range for an 8-year-old — with slow-frequency dominance consistent with the attentional and executive profile. Following intervention, mean EEG frequency increased to 5.86–8.3 Hz overall, reaching 8.3 Hz in posterior regions, indicating a shift toward a more mature, faster-dominant spectral composition. This change was accompanied by a well-defined anterior-to-posterior frequency gradient, which was less organized pre-intervention. No deviations from normative references were detected post-intervention in broadband or narrowband spectral analyses, except for a mild and circumscribed increase in spectral energy in the 10.16–10.55 Hz sub-band.

**Fig. 7:**
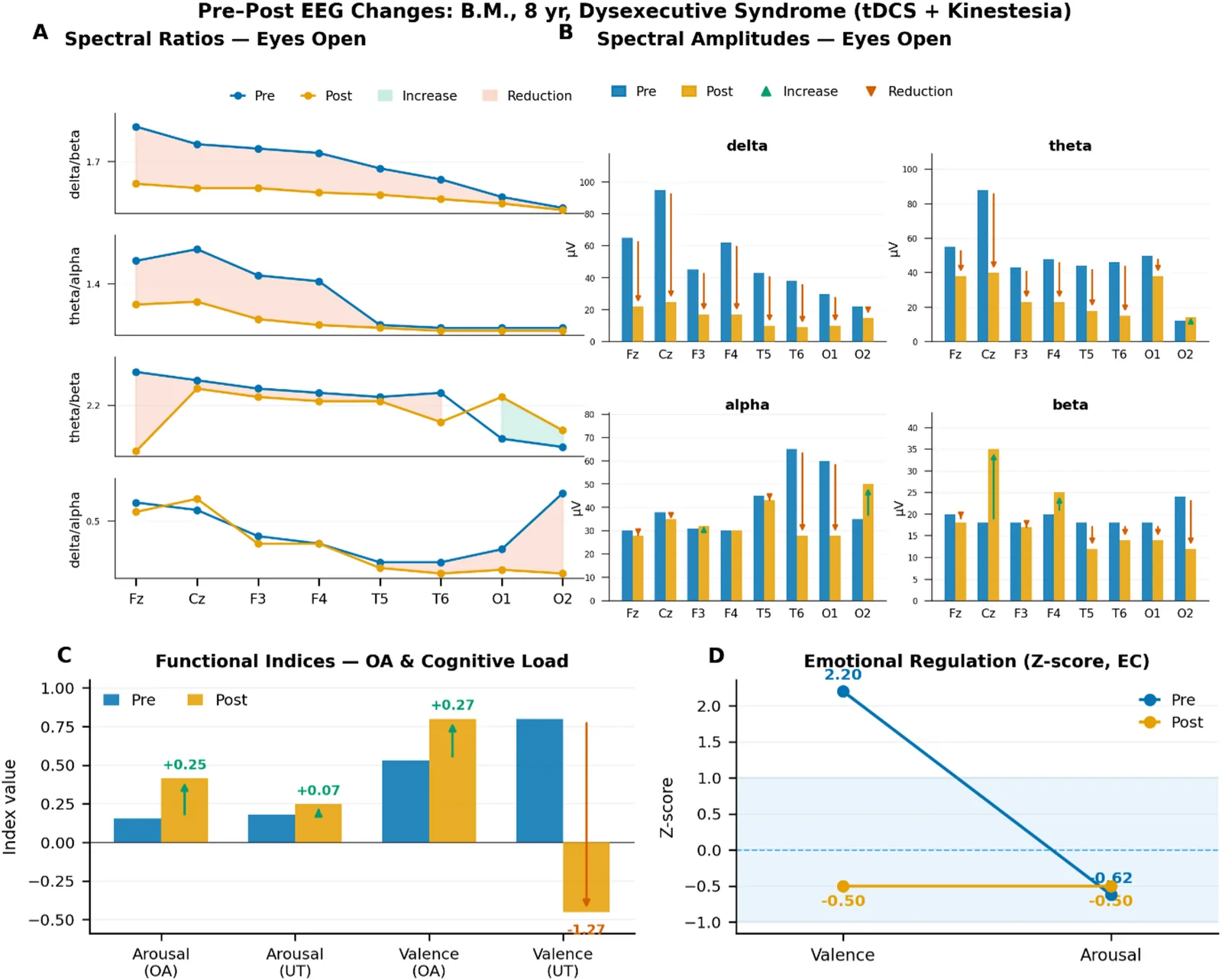
Pre–post neurorehabilitation EEG changes in a representative clinical case (B.M., 8 years, dysexecutive syndrome). Quantitative EEG was recorded before and after the therapeutic intervention under eyes-open (OA) and cognitive load (UT) conditions. Blue: pre-intervention; orange: post-intervention. (**A**) Spectral ratios — eyes open. Pre–post trajectories of four log-ratio indices (delta/beta, theta/alpha, theta/beta, delta/alpha) across eight electrodes (Fz, Cz, F3, F4, T5, T6, O1, O2). Green shading indicates post > pre (increase); red shading indicates post < pre (reduction). Consistent reductions in slow-to-fast ratios are observed across frontal and central sites, with the largest changes in theta/beta and delta/beta. (**B**) Spectral amplitudes — eyes open. Absolute spectral power (µV) pre and post for delta, theta, alpha, and beta bands across the same electrode set. Arrows indicate the direction of change at each electrode; orange arrows indicate reduction (post < pre), green arrows indicate increase (post > pre). Marked reductions in delta and theta amplitude are observed at frontal and central sites (notably Cz and F3), alongside selective beta increases at Cz and F4. (**C**) Functional state indices. Pre–post bar comparison of the Arousal and Valence indices under OA and UT conditions. Arrows indicate the magnitude and direction of change (Δ). (**D**) Emotional regulation Z-scores (eyes-closed condition). Pre–post comparison of Valence and Arousal expressed as normative Z-scores derived from the age-matched models developed in this study. The shaded band indicates the ± 1 SD normative range (Z = ± 1). Pre-intervention Valence deviated markedly from the norm (Z = + 2.20), returning to within the normative range post-intervention (Z = − 0.50). Arousal showed a smaller but consistent shift toward normalization. The dashed line marks Z = 0 (population mean)

Figure 7A and B illustrate electrode-level spectral ratios (delta/beta, theta/alpha, theta/beta, delta/alpha) and absolute spectral amplitudes in the eyes-open condition. Consistent reductions in slow-to-fast ratios were observed post-intervention across frontal and central sites, with the largest changes in theta/beta and delta/beta. Absolute spectral amplitudes confirmed this pattern: marked reductions in delta and theta were observed at nearly all electrode sites, particularly prominent at Cz and frontal locations (delta: Cz pre ≈ 95 µV, post ≈ 25 µV; theta: Cz pre ≈ 85 µV, post ≈ 40 µV). Beta amplitude showed selective increases at Cz and F4, compatible with enhanced frontocentral activation. The Z-score theta/beta ratio at F3 decreased from 0.48 to 0.03 (− 93.7%) and at F4 from 0.47 to 0.30 (− 36.2%), compatible with normalization of frontal attentional regulation indices (Arns et al. 2013; Ogrim et al. 2012). The individual alpha peak frequency increased from 9.38 to 9.77 Hz (+ 4.1%), compatible with accelerated thalamocortical maturation.

##### Functional State Indices and Z-Score Normalization

Figure 7C shows the pre–post comparison of functional state indices under eyes-open and cognitive load conditions. Substantial increases in Arousal (+ 0.25) and Valence (+ 0.27) were observed in eyes-open condition, compatible with enhanced cortical activation and hemispheric asymmetry reorganization. Under cognitive load, Arousal increased moderately (+ 0.07), while the Valence index showed a pronounced reduction (− 1.27), compatible with a shift toward right-hemisphere dominance under executive demand — a pattern compatible with reduced frontal cognitive overload following the intervention.

Figure 7D shows the pre–post Z-score comparison of Valence and Arousal in the eyes-closed condition, anchored to the age-matched normative models developed in this study. The Valence index shifted from Z = + 2.20 pre-intervention to Z = − 0.50 post-intervention — a reduction of more than 2.7 standard deviations into the normative range — consistent with normalization of pathological left-hemisphere alpha dominance. The Arousal index showed a parallel shift from Z = − 0.62 to Z = − 0.50, remaining within the normative band throughout but moving toward the population mean. These shifts in the eyes-closed condition are complemented by the broader pattern of functional index changes observed across states: in the eyes-closed condition, the Valence index had shown a marked deviation of Z = − 2.82 pre-intervention, returning to Z = − 0.61 post-intervention, while the Arousal index decreased by 0.10, both compatible with a generalized normalization of the neurophysiological state at rest

These results are clinically corroborated by the expert neurologist’s assessment, which noted improved organization of the resting EEG, a stable and well-organized posterior alpha rhythm, and only mild intermittent disturbance of frontal-temporal activity — a substantially improved profile relative to the pre-intervention recording. The quantitative indices provided, in this case, an objective, reproducible complement to visual inspection, anchoring the observed changes within a normative probabilistic framework and enabling their direct comparison against age-expected population distributions.

### The Need for Multi-State Normative References

This case also highlights a methodological gap that the present framework only partially addresses. The most clinically informative changes were observed not only in the eyes-closed resting condition—for which normative models are available—but in the EO and CL conditions (Fig. 5A and B). The reduction in theta/beta ratio under cognitive load, the increase in beta amplitude at frontal sites during EO, and the modulation of Arousal and Valence indices across conditions all constitute evidence of improved neurophysiological function that cannot currently be anchored to age-expected norms, because lifespan normative references for EO and cognitive activation conditions remain unavailable. This observation reinforces the call for future normative databases incorporating multi-state recording protocols (see Sect.  4.5.1) and underscores that the clinical interpretability of qEEG as a therapeutic monitoring tool depends critically on the availability of condition-specific normative references.

## Discussion

The present study contributes to the ongoing revival of quantitative EEG by embedding a clinically relevant family of derived spectral indices within age-dependent probabilistic normative frameworks. The resulting models enable individualized inference—answering not merely whether brain activity is atypical, but by how much and in which direction relative to the population norm for a given age. In doing so, this work revisits the original vision of neurometrics while integrating the computational and data-harmonization advances of modern neuroscience.

### From Qualitative Heuristics to Normative Precision

For decades, clinical EEG practice has relied on expert visual inspection to identify paroxysmal activity, focal slowing, or diffuse abnormalities. While this remains essential for the detection of unambiguous pathology, it systematically discards the wealth of information encoded in quantitative spectral features. The neurometrics framework proposed by John et al. (John 1977; John et al. 1988) articulated a compelling alternative: brain function follows predictable statistical trajectories across the lifespan that can be modeled, and departures from these trajectories can be expressed as probabilistic scores amenable to clinical interpretation.

The present results demonstrate that this principle extends naturally to derived spectral ratios and composite functional indices. A theta/beta ratio reported as a raw value has no intrinsic clinical meaning; the same value expressed as a Z-score of + 2.5 relative to an age-matched normative distribution immediately communicates a meaningful deviation, with a probability of occurrence below 1.2% in a neurotypical population of that age. This translation from measurement to inference is what distinguishes a normative biomarker from a descriptive metric, and it is precisely the translation that routine clinical reports currently fail to provide (Ko et al. 2021; Prichep 2005).

### Functional Indices as Evidence of Therapeutic Efficacy

One of the most clinically significant contributions of this framework lies in the extension of normative modeling beyond simple spectral power to functional indices of arousal, emotional valence, and cognitive engagement. These composite metrics have been repeatedly linked to neuropsychiatric conditions and to the outcomes of therapeutic interventions: theta/beta elevation in attentional dysregulation (Arns et al. 2013; Ogrim et al. 2012), frontal alpha asymmetry in mood disorders and affective valence (Davidson 1998; Smith et al. 2017), engagement index reduction in fatigue and inattention (Pope et al. 1995), and arousal index perturbations in vigilance disorders (Hegerl et al. 2012; Olbrich et al. 2015).

However, the clinical utility of these indices has been severely constrained by the absence of normative reference values. Without such references, pre–post comparisons in therapeutic monitoring yield only directional information—the index increased or decreased—without any indication of whether the change represents movement toward, arrival at, or departure from normotypical function. The normative Z-score framework resolves this ambiguity by providing a principled measure of the direction and magnitude of neurophysiological change relative to the population distribution. This constitutes a qualitative upgrade in the evidential value of longitudinal qEEG monitoring—from a descriptive record of change to a quantitative index of normalization—that is directly relevant to evidence-based neurorehabilitation (Kropotov 2009, 2016; Collura et al. 2010).

### Electrode-level Spectral Normalization: A Universal and Principled Preprocessing Step

A methodological contribution of this work is the adoption of Electrode-level Spectral Normalization (ESN) as a universal preprocessing step applied to each electrode’s spectral values before index computation. Technical variability between EEG acquisition systems—arising from differences in amplifier characteristics, electrode impedance standards, reference schemes, and digital filtering—constitutes a well-documented source of systematic variance in spectral estimates that is unrelated to neurophysiology (Li et al. 2022). At the same time, individual differences in scalp and skull conductivity introduce subject-specific multiplicative scale factors that affect all electrodes simultaneously. Both sources of variability are multiplicative on the power scale and therefore appear as additive offsets in log-transformed spectral values—precisely the scale on which normative indices are computed.

**Potential limitations of ESN and situations where it may be contraindicated (see also Supplementary Fig. 11 for a proof-of-concept simulation).**

By design, ESN removes multiplicative amplitude differences across electrodes, making it specifically appropriate when the clinical question concerns the relative spectral composition — i.e., how power is distributed across frequencies and electrodes — rather than absolute amplitude levels. This property is well suited for ratio-based indices such as TBR, TAR, DBR, Arousal, and Valence, where inter-individual and inter-device differences in absolute gain would otherwise confound normative comparisons.

However, ESN is contraindicated when absolute EEG power is itself the primary biomarker of interest — specifically, in conditions where the spectral pattern is relatively preserved but overall amplitude is globally suppressed, such as pharmacological burst-suppression or deep coma with near-isoelectric EEG. In such cases, ESN normalization would absorb the amplitude suppression and yield apparently normal spectral Z-scores despite severe pathology. In these contexts, raw power spectra or globally-referenced metrics that preserve absolute amplitude information are more appropriate. Clinicians applying ESN-normalized normative Z-scores to populations with suspected global amplitude suppression should interpret results with caution. Notably, ESN operates per-electrode rather than applying a single global scalar, thereby correcting for spatially non-uniform impedance differences — a confound that would otherwise affect inter-electrode ratio indices even when overall recording gain is matched across devices. A proof-of-concept simulation illustrating these trade-offs is provided in Supplementary Fig. 12.

It should also be noted that the 9 recording devices included in this database may not encompass the full diversity of clinical EEG equipment encountered in practice. Kruskal-Wallis tests across devices were statistically significant for all indices (H(8) range: 17.85–47.07, all *p* < 0.05), reflecting the high statistical power of the full sample rather than clinically meaningful differences; effect sizes were negligible (η² range: 0.008–0.031) and maximum mean device deviations remained within |Z| < 0.75 (Supplementary Fig. 10; Supplementary Table 4). External validation with additional equipment is warranted before applying these norms to devices not represented in the normative database.

### Relation to the Normative Modeling Tradition in Neuroscience

The present work aligns with a broader movement in computational neuroscience toward normative modeling as a framework for understanding individual variability in brain measurements (Marquand et al. 2016). Marquand et al. (2016) articulated the key insight that heterogeneous clinical populations cannot be understood through group-mean comparisons alone but require individual-level deviation scores relative to a well-characterized healthy distribution. Subsequent applications of this paradigm to structural MRI—including the large-scale lifespan brain charts of Bethlehem et al. (Bethlehem et al. 2022)—have demonstrated its value for identifying individuals whose brain measurements deviate from typical developmental trajectories, independently of diagnostic category.

Quantitative EEG is particularly well-suited for this approach. Its low cost, temporal resolution, and widespread clinical availability make it a practical complement to MRI-based normative atlases, extending the reach of normative modeling to settings where structural or functional neuroimaging is unavailable. The HarMNqEEG database (Li et al. 2022), with its multinational harmonized design and lifespan coverage, provides a foundation for qEEG normative modeling that is broadly comparable in scope—if not yet in spatial resolution—to the large neuroimaging datasets underlying MRI brain charts.

The spectral normative framework developed here is complementary to the microstate-based normative tradition pioneered by Koenig, Michel, and colleagues. EEG microstates—quasi-stable topographic configurations of the scalp potential field—follow systematic age-dependent trajectories across the lifespan (Koenig et al. 2002), correlate robustly with large-scale resting-state fMRI networks (Rajkumar et al. 2021), and show sensitivity to neurological and psychiatric conditions, establishing them as a normative framework at the level of global field dynamics (Michel and Koenig 2018). The two frameworks address orthogonal dimensions of spontaneous brain activity: momentary global field topography (microstates) versus frequency-domain composition (spectral ratios). Both have converged on the same normative imperative—individual measurements acquire clinical meaning only when anchored to age-expected population distributions (Koenig et al. 2002; Marquand et al. 2016; Bethlehem et al. 2022)—and future work integrating both within a unified lifespan reference would provide a substantially richer normative atlas of human brain electrophysiology.

#### EEG Reference and Its Implications for Normative Modeling

The HarMNqEEG database employs the common average reference, consistent with most large-scale normative EEG databases and standard clinical qEEG practice. An alternative reference scheme with theoretical advantages is the Reference Electrode Standardization Technique (REST; Yao 2001), which approximates the potential at infinity and may reduce reference-related distortions in scalp topographies. While reference choice can in principle influence the values of electrode-level spectral indices, its practical impact on the present normative framework is limited for three reasons. First, ratio-based indices computed within the same electrode (TBR, TAR, DBR, ABR) are generally less sensitive to reference choice than absolute power measures, particularly when the reference transformation affects frequencies similarly within an electrode. Second, ESN normalization removes multiplicative amplitude scaling within each electrode, rendering it robust to reference-induced amplitude offsets that are approximately uniform across frequency. Third, because both the normative database and clinical recordings referred to these norms share the same reference convention, systematic reference effects are reduced in the Z-score computation. Users applying these norms to recordings acquired under a different reference scheme — including REST — should apply an appropriate re-referencing step before index computation. Formal evaluation of reference effects on normative Z-scores across schemes remains an important direction for future work.

#### Classical Spectral Band Indices and Periodic/Aperiodic Decomposition: Complementary Frameworks

**Mathematical equivalence of spectral frameworks.** The formal separation of EEG spectral components into periodic and aperiodic contributions was introduced by Pascual-Marqui et al. (1988) in the ξ–α model, which represents the power spectral density (PSD) as the superposition of two physiologically interpretable processes: S(ω) = S_ξ_(ω) + S_α_(ω), where the aperiodic component ξ follows a Matérn spectral density S_ξ_(ω) = A_ξ_ / (κ_ξ_^2^ + ω^2^)^ν+½^, with amplitude A_ξ_, inverse length-scale κ_ξ_ and smoothness order ν; and the periodic component α follows a Hida–Matérn (generalized Lorentz) profile S_α_(ω) = A_α_ / [1 + ((ω − U_α_) / B_α_)^2^]^60^, centered at peak frequency U_α_ with bandwidth B_α_. In the high-frequency limit and on a log–log scale, the Matérn density reduces to the familiar power law S_ξ_(ω) ∝ 1/ω^β^ with β = 2ν + 1, which is precisely the “aperiodic exponent” estimated by FOOOF/specparam (Donoghue et al., 2020) and related tools. A recent comprehensive analysis (García Reyes et al., 2026) formally demonstrated — through a systematic review of all major spectral component models (Zetterberg, FOOOF, SPRiNT, PAPTO, IRASA, and ξ–αNET) — that **every current periodic/aperiodic decomposition framework approximates the aperiodic component as a Matérn process or one of its limiting cases.** This mathematical unification carries a direct epistemological consequence: the aperiodic component estimated by any of these methods and the ξ component of the classical ξ–α model are the same mathematical object, differing only in parameterization and frequency-axis scaling. The claim that periodic/aperiodic decomposition constitutes a fundamentally superior or categorically distinct approach to classical spectral analysis is therefore not supported by the underlying mathematics — both frameworks are instances of the same family of Matérn spectral representations.

**Parametric versus nonparametric spectral decomposition.** The mathematical equivalence established above applies strictly to the parametric family of spectral decomposition methods, all of which presuppose a specific functional form—typically Matérn or Hida–Matérn—for the aperiodic and periodic components. This equivalence does not extend to nonparametric approaches. The ξ–π model (Hu et al. 2024) represents a methodologically distinct class: rather than fitting a prespecified parametric form, it estimates the spectral density via nonparametric basis expansion, allowing the data to determine the spectral profile without the constraints of a fixed parameterization. While this sacrifices the direct mechanistic interpretability of parameters such as the aperiodic exponent or peak frequency, it gains flexibility in capturing spectral shapes that may deviate from the Matérn assumption. A rigorous formal comparison between parametric (Matérn-family) and nonparametric (ξ–π) spectral decomposition, including their relative robustness to model misspecification and their differential utility for normative modeling, remains an important direction for future work.

**Complementarity rather than competition.** Conventional band-ratio indices such as TBR, TAR, and DBR integrate both the ξ and α components within each frequency band; changes in the aperiodic exponent β or in the peak frequency U_α_ can in principle contribute to age-related variations in these ratios. This is not a limitation unique to band indices — it reflects the fact that any scalar spectral summary (including the aperiodic exponent itself) integrates a mixture of both components, because the Matérn and Hida–Matérn processes overlap across the entire frequency axis. The practical advantage of explicit decomposition lies in disentangling these contributions for mechanistic interpretation, not in accessing a different underlying signal. Moreover, no established clinical indices analogous to TBR, Arousal or Valence are currently defined in terms of periodic/aperiodic decomposition parameters, limiting the immediate applicability of this framework for normative Z-score computation.

**Lifespan trajectories and extensions of this work.** García Reyes et al. (2026) further demonstrated, using the same HarMNqEEG database analyzed here and the full ξ–αNET model (which incorporates anatomical connectivity, interareal conduction delays and Bayesian source imaging), that the aperiodic component follows a monotonic lifespan decrease while the periodic α component shows a growth-and-decline trajectory — findings that are directly interpretable within the Matérn parameterization of the ξ–α model. Future extensions of this normative framework may incorporate explicit periodic/aperiodic decomposition to provide normative references for aperiodic exponent β and offset A_ξ_, and for peak frequency U_α_, alongside the classical spectral ratio indices reported here — constituting complementary rather than competing normative references.

### Limitations and Future Directions

Several limitations of the present work merit explicit acknowledgment. First, inter-site technical variability is addressed through ESN rather than Riemannian harmonization of the cross-spectral matrices (Li et al. 2022). This choice is deliberate: Riemannian harmonization operates on the full cross-spectral tensor and efficiently removes site-level biases in the covariance geometry, but it introduces cross-electrode mixing that removes the direct neurophysiological interpretation of per-electrode spectral ratios and asymmetry indices. ESN corrects for multiplicative amplitude offsets while preserving the electrode-level meaning of each index. Riemannian harmonization remains a valuable approach for applications where the full covariance structure is the object of interest, such as source imaging and functional connectivity.

Second, the present normative models were derived exclusively from resting-state spectral features and do not incorporate source-level information. The integration of electromagnetic source imaging methods such as VARETA (Bosch-Bayard et al. 2001) and sLORETA (Pascual-Marqui 2002) within a normative framework would allow the localization of deviating generators, adding a spatial dimension to the probabilistic biomarker approach. This extension is technically feasible within the HarMNqEEG infrastructure and constitutes a natural next step toward a comprehensive normative atlas of human brain electrophysiology.

Third, the normative sample spans 5–90 years (*N* = 1,564) but is unevenly distributed across the lifespan, with 70% of participants falling in the 10–40 year range (*n* = 1,089). The extreme age groups are more sparsely represented (< 10 year, *n* = 172; ≥ 70 year, *n* = 141; Supplementary Fig. 14). Although the P-spline smoothers used in GAMLSS are regularized and resistant to overfitting in data-sparse regions, normative Z-scores at the age extremes should be interpreted with additional caution. Expansion of the normative database at these age ranges — particularly adults over 70 years and children under 10 years — remains an important direction for future work.

Fourth, the clinical validation of these normative indices as diagnostic or prognostic biomarkers in specific neurological and psychiatric conditions—ADHD, mood disorders, mild cognitive impairment, epilepsy—requires prospective studies comparing Z-score profiles against gold-standard clinical diagnoses and longitudinal outcomes. The normative models presented here provide the infrastructure for such studies; their clinical sensitivity and specificity remain to be established across patient populations.

The present normative framework addresses the population-referenced deviation question (how does this individual compare to healthy peers of the same age? ) and is complementary to two emerging lines of research that address distinct clinical questions. First, EEG brain age prediction: Hu et al. (2025a, b) applied GAMLSS-based lifespan modeling to the same HarMNqEEG database to derive scalar biological age estimates from EEG, demonstrating that the database supports multiple normative applications simultaneously. Scalar brain age and population-referenced Z-scores are mutually reinforcing rather than redundant: brain age collapses deviation across all indices into a single biomarker, while Z-scores preserve the index-level and electrode-level granularity needed for mechanistic interpretation and targeted intervention. Second, supervised machine-learning classification: two recent papers address this problem from complementary perspectives. Hu et al. (2026) proposed GCAF-Net, a geometry-informed cross-attention fusion graph neural network integrating functional connectivity, spectral features, and electrode spatial topology for EEG-based AD/FTD/HC classification, achieving 97% accuracy on the ds004504 dataset and 98.69% on the independent BrainLat dataset. Independently, Mlinarčič et al. (2026) demonstrated classification of Alzheimer’s disease, frontotemporal dementia, and healthy controls from resting-state EEG using complementary methods. Supervised classification and the normative Z-score framework address distinct clinical scenarios: classification assigns a categorical label from a predefined set, whereas Z-scoring quantifies the degree and pattern of individual deviation without presupposing a diagnostic category, making it suitable for heterogeneous populations and longitudinal monitoring where group identity is unknown or evolving.

#### Eyes-closed Resting State as the Sole Normative Condition, and a Proposed Protocol for Future Expansion

The HarMNqEEG database is exclusively restricted to eyes-closed resting-state recordings. While this condition provides the most stable and reproducible baseline for normative reference, it necessarily leaves uncharacterized a clinically important dimension of EEG dynamics: the brain’s response to external stimulation and cognitive demand. Alpha reactivity upon eye opening — a robust index of thalamocortical integrity (Pfurtscheller and Lopes da Silva 1999; Fonseca et al. 2011) — and electrophysiological changes during mental arithmetic, working-memory tasks, or hyperventilation may reveal abnormalities that are invisible at rest. Certain EEG patterns, including impaired arousal modulation and executive dysregulation, emerge only under cognitive demand (Swingle 2015; Fairclough et al. 2024).

To guide future normative expansion, we propose a short sequential protocol that establishes a graded continuum from resting baseline to executive load, using only verbal instructions and no additional materials: (1) Eyes closed, 2–3 min — thalamocortical baseline; (2) Eyes open, 1–2 min — alpha reactivity and EC→EO modulation; (3) Backward counting from 100 by 1s, 30–60 s — sustained attention; (4) Serial subtraction by 7s, 30–90 s — cognitive load and working memory; (5) Backward digit span, 1–2 min — working-memory manipulation; (6) Covert verbal fluency, 30–60 s — lexical retrieval and executive search (see Table 1). Tasks involving overt speech should be avoided due to electromyographic contamination. This protocol is consistent with recommendations in the clinical EEG biofeedback literature (Swingle 2015) and supported by evidence on EEG workload markers (Borghini et al. 2014; Onton et al. 2005). Normative lifespan references for activation conditions remain largely unavailable and represent a priority for future database development.

**Table 1:** Brief cognitive challenge tasks that can be integrated into a resting-state qEEG protocol, ordered by increasing cognitive demand. Tasks are proposed as a future extension of eyes-closed normative protocols to capture EEG dynamics under graded activation conditions

Task	Domain probed	Typical instruction	Duration	Main advantages	Limitations / artifacts	Clinical utility in qEEG
Eyes open (EO)	Arousal/alpha reactivity	“Eyes open, look straight ahead.”	1–3 min	No materials; standardized	Eye blinks, saccades	Alpha reactivity; EC→EO modulation (Barry and De blasio, 2017)
Backward counting	Sustained attention, executive control	“Count backward from 100 to 1.”	30–60 s	Easy; scalable; no materials	Overt speech adds EMG; use covert	Practical challenge; recommended by Swingle/ClinicalQ (Swingle 2015)
Serial subtraction	Cognitive load, working memory, inhibitory control	“Subtract 7s from 100 mentally.”	30–90 s	Strong EEG workload literature; adjustable difficulty	Overt response contaminates the EEG; arithmetic skill confound	Best no-equipment workload task for brief EEG blocks (Fairclough et al. 2024)
Backward digit span	Working memory manipulation	“Repeat these digits backwards.”	1–2 min	Well-defined executive demand; adaptable	Examiner timing required; overt artifacts	Frontal-executive probe; sensitive to WM capacity (Onton et al. 2005)
Covert verbal fluency	Lexical retrieval, executive search	“Think of as many animals as possible.”	30–60 s	No materials; language/executive load	Compliance harder to verify if fully covert	Frontal/language network assessment (Borghini et al. 2014)

EC = eyes closed; EO = eyes open; EMG = electromyographic artifact; WM = working memory. Covert or minimal-response variants are preferred to minimize signal contamination. References in brackets correspond to citations in the main text

## Conclusions

Reclaiming quantitative EEG as a primary diagnostic and monitoring instrument is not a nostalgic return to the past, but a scientifically grounded aspiration for modern precision neurophysiology. The normative models presented here — for spectral power ratios, cognitive-emotional state indices, and physiological parameters — provide the statistical infrastructure required to transform descriptive EEG metrics into probabilistic reference values. By anchoring individual measurements to age-adjusted population distributions and expressing deviations as Z-scores under the unified ESN-normalized framework, the present work offers clinicians and researchers a principled basis for asking: Is this individual’s brain activity typical for their age? Has an intervention produced a neurophysiologically meaningful change toward normotypical function? Answering these questions in clinical practice will require prospective validation in patient populations, which this framework is now positioned to support.

The use of GAMLSS modeling captures the non-linear developmental trajectories of EEG indices across the lifespan and appropriately represents age-dependent changes in both expected values and inter-individual variability — properties that earlier normative approaches based on fixed-variance linear models could not accommodate. The ESN normalization strategy ensures that the framework remains applicable across the full spectrum of clinical recording contexts, from well-documented research-grade EEG to legacy or minimally documented clinical files, without sacrificing methodological transparency.

To our knowledge, this represents the first systematic effort to construct harmonized, multinational normative models for this family of derived functional qEEG indices. The work establishes the foundational normative layer for eyes-closed resting-state conditions. Future extensions — incorporating eyes-open, cognitive activation, hyperventilation, and photic stimulation conditions, and integrating source-level normative mapping — will complete the transition from static to functional neurometrics and fulfill the original promise of quantitative EEG as an objective, individualized tool for evidence-based neurorehabilitation. The clinical sensitivity and specificity of these normative Z-scores as diagnostic or prognostic biomarkers remain to be established in prospective patient studies and constitute the natural next step for this line of research.

## Supplementary Information

Below is the link to the electronic supplementary material.

## Data Availability

The HarMNqEEG normative database is publicly available at https://github.com/CCC-members/HarMNqEEG. GAMLSS model parameters, centile curves, and Z-score computation scripts (MATLAB/R) will be released at https://github.com/oldgandalf/Normatives-spectral-based-indexes-from-EEG-resting-state upon acceptance, subject to a reasonable request to the corresponding author.

## Declarations

### Artificial Intelligence

The authors used Claude (Anthropic, claude.ai) to assist with the following tasks during manuscript preparation: Python scripting for statistical analysis and figure generation, editing and revision of manuscript text for clarity and concision, and formatting of the reference list. All analytical decisions, interpretation of results, and final manuscript content were made by and remain the responsibility of the authors. The use of this tool was reviewed and approved by all co-authors.
