# Supplementary material for: Back to the Future of qEEG: Lifespan Normative Modeling of Spectral Ratios and Functional Indices with Potential Applications to Therapeutic Monitoring
Source: Brain Topogr. 2026 Jul 31;39(5):87. doi: 10.1007/s10548-026-01240-4 (PMC13427868; doi:10.1007/s10548-026-01240-4)
Supplement: Supplementary file 1 — Supplementary Material 1 [file 10548_2026_1240_MOESM1_ESM.docx]

# Supplementary Figures

Age-dependent normative centile curves for different indices and ratios across all 19 electrodes of the 10–20 system. Each panel corresponds to one electrode, arranged in approximate anatomical order from anterior (top) to posterior (bottom). Individual observations from the HarMNqEEG normative database (n = 1,564; ages 5–97 years) are shown as grey circles. The solid red line indicates the normative mean (μ) estimated by GAMLSS with P-spline smoothers on log(age). The shaded grey band represents the ±1.96σ normative interval, corresponding to the 2.5th–97.5th centile range under the fitted conditional distribution. Dashed lines mark the outer ±1.96σ bounds. The x-axis is displayed on a logarithmic scale.


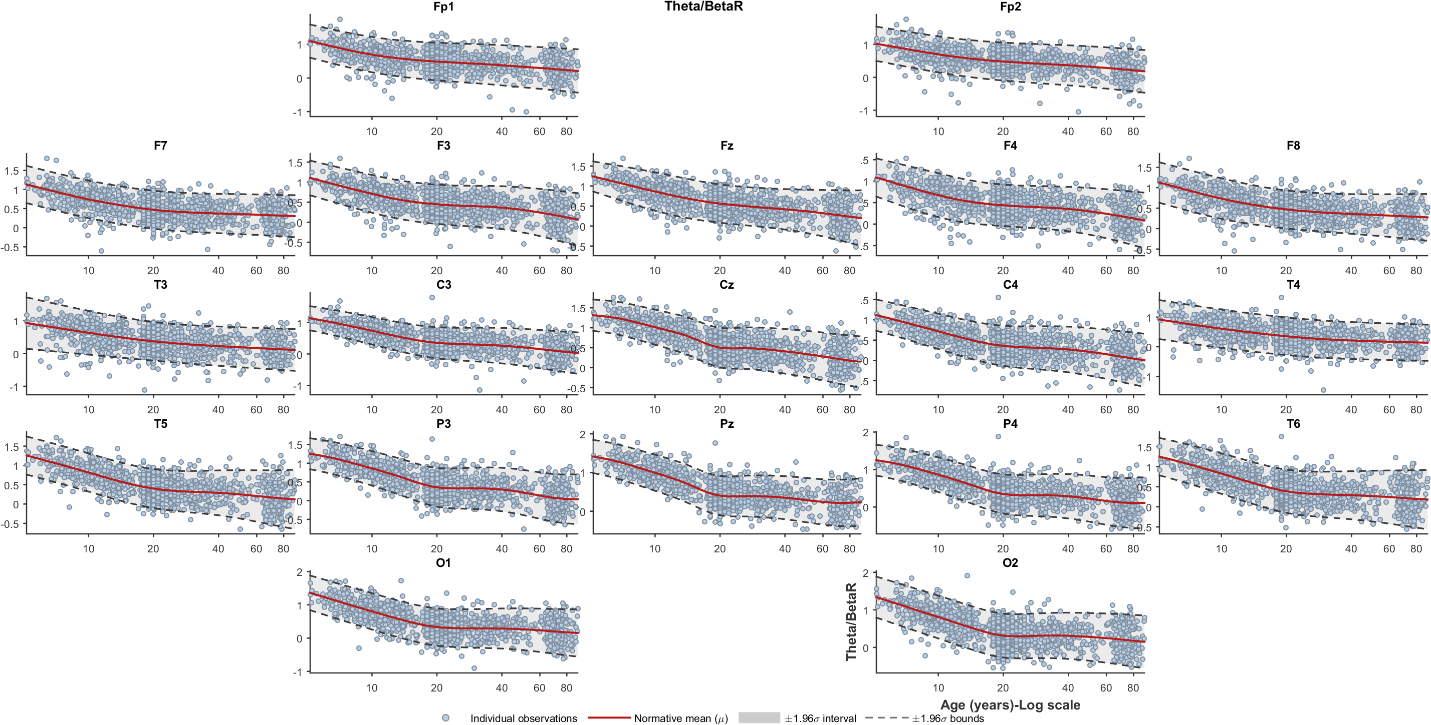


**Sup. Figure 1.** Theta/Beta Ratio (TBR), defined as ln[θ(ch)] − ln[β(ch)] at each electrode ch. The consistent age-dependent decrease observed across all electrodes — most pronounced at frontal and central sites — reflects progressive cortical maturation and reduction of slow oscillatory dominance from childhood through adulthood (Arns et al. 2013; Ogrim et al. 2012).


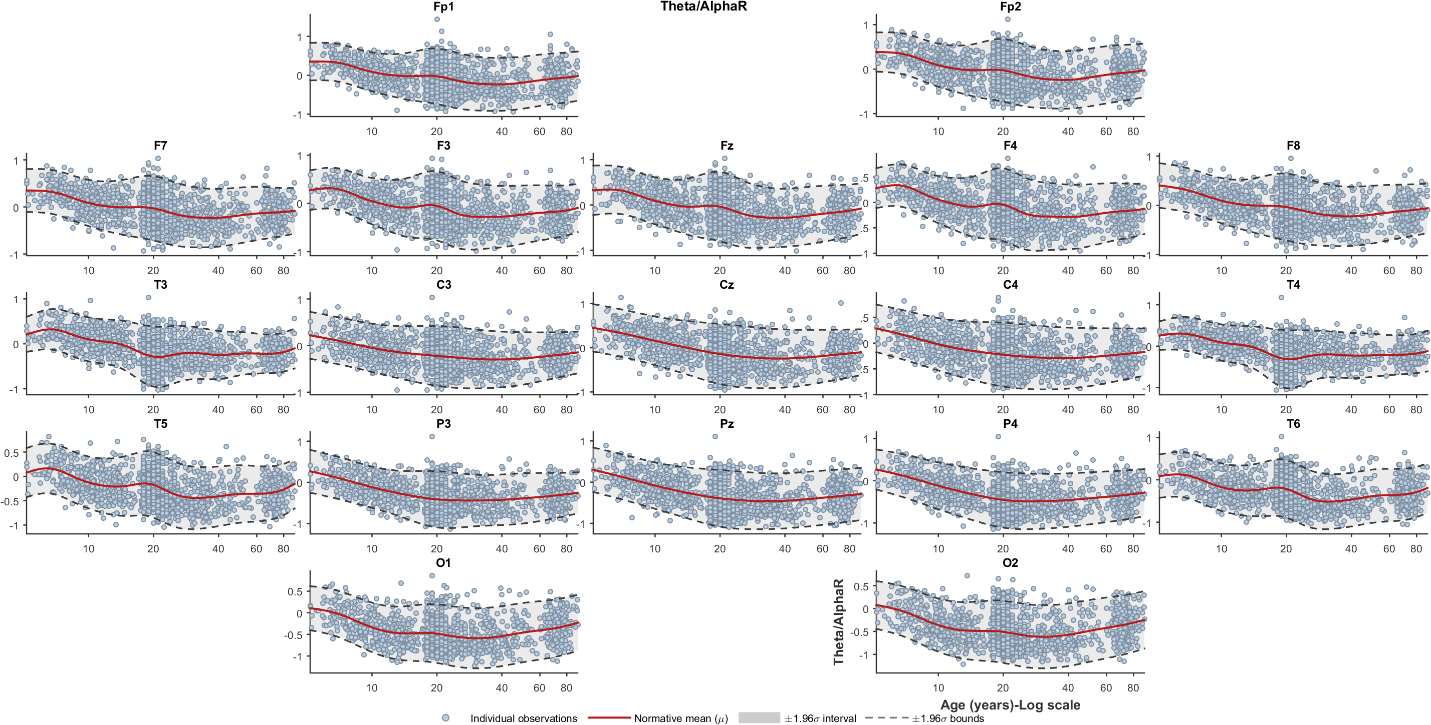


**Sup. Figure 2.** Theta/Alpha Ratio (TAR), defined as ln[θ(ch)] − ln[α(ch)] at each electrode ch. The age-dependent decrease is consistent across all electrodes, reflecting the progressive strengthening of alpha relative to theta activity during cortical maturation. The trajectory is less steep than for TBR, and the normative band is narrower in posterior regions — where alpha dominance is more consistent across individuals — compared to frontal sites, where greater inter-individual variability in vigilance-related theta modulation broadens the normative interval (Klimesch 1999).


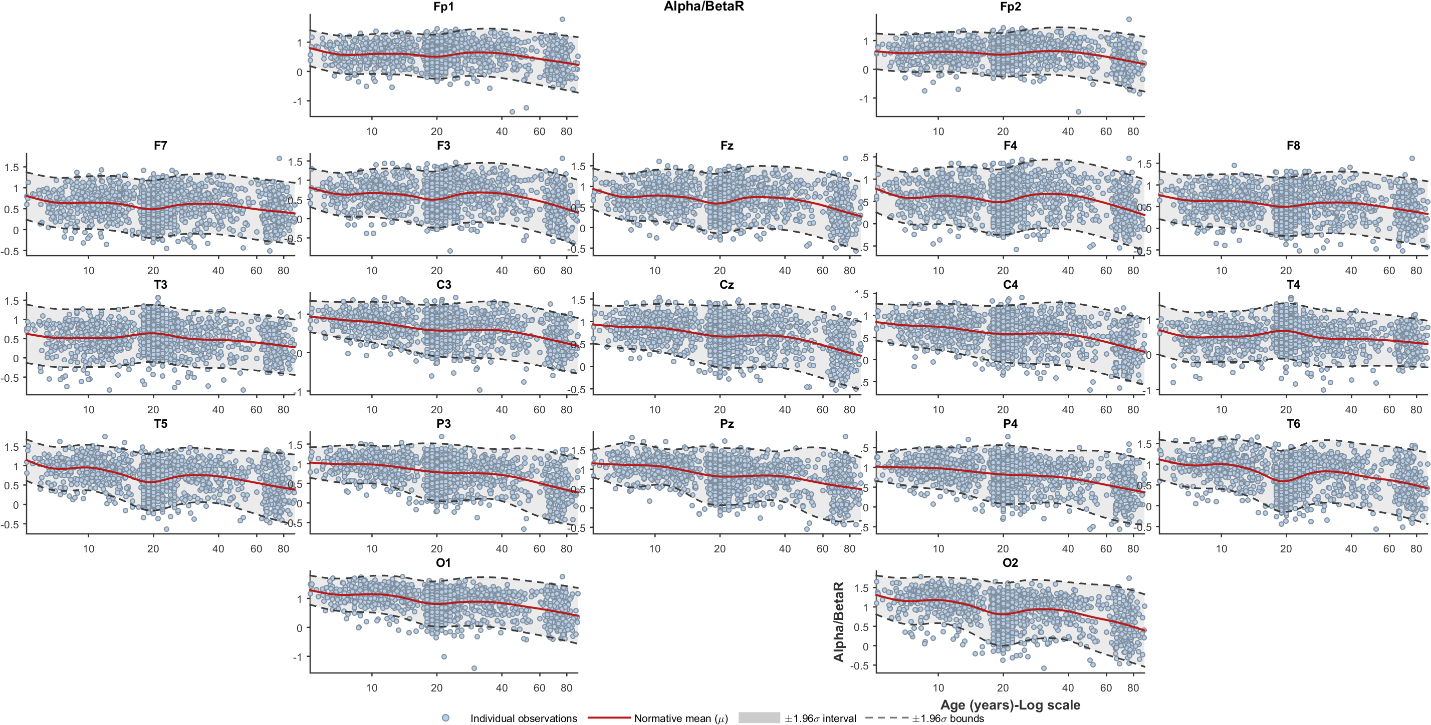


**Sup. Figure 3.** Alpha/Beta Ratio (ABR), defined as ln[α(ch)] − ln[β(ch)] at each electrode ch. In contrast to TBR and TAR, ABR shows a non-monotonic developmental trajectory in several electrodes: values increase during childhood and adolescence — reflecting the well-documented strengthening of alpha oscillations during cortical maturation — before stabilizing or modestly declining in adulthood. This pattern is most pronounced at posterior and parietal sites (P3, Pz, P4, O1, O2), where alpha dominance is strongest, and attenuated at frontal sites where beta activity contributes more substantially to the ratio. The normative band is notably wider at frontal electrodes, reflecting greater inter-individual variability in the alpha/beta balance in prefrontal regions.


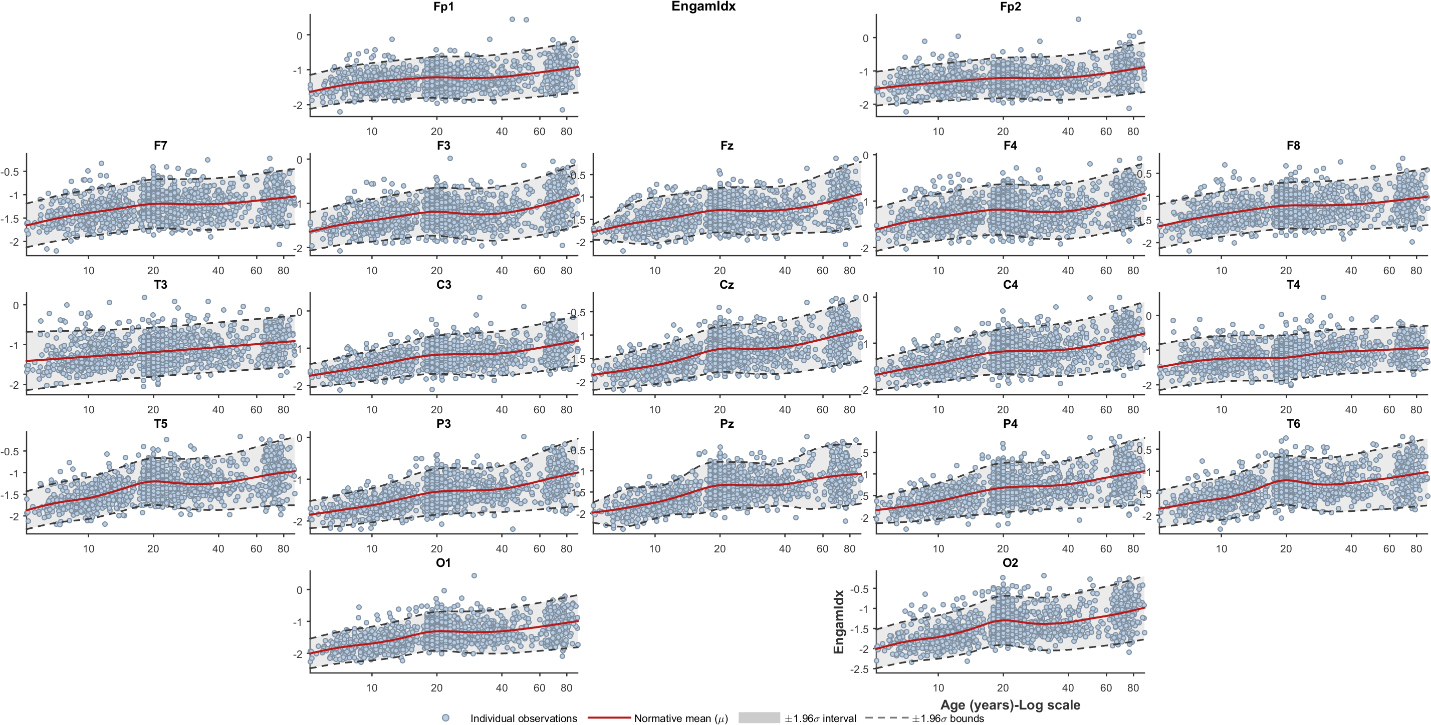


**Sup. Figure 4.** Engagement Index (EI), defined as EI = ln[β̄] − ln[ᾱ + θ̄], where the overbar denotes the mean over all 19 electrodes; the log-ratio formulation yields the negative values observed, as alpha and theta power jointly exceed beta power across the lifespan under resting eyes-closed conditions. The normative trajectory shows a gradual increase with age across all electrodes, consistent with a progressive shift toward relatively greater beta activity — reflecting increasing cortical activation efficiency and reduced slow-wave dominance — from childhood through adulthood (Pope et al. 1995). Inter-individual variability is largest in childhood and decreases progressively with age, resulting in a narrowing normative band in adults and older adults.


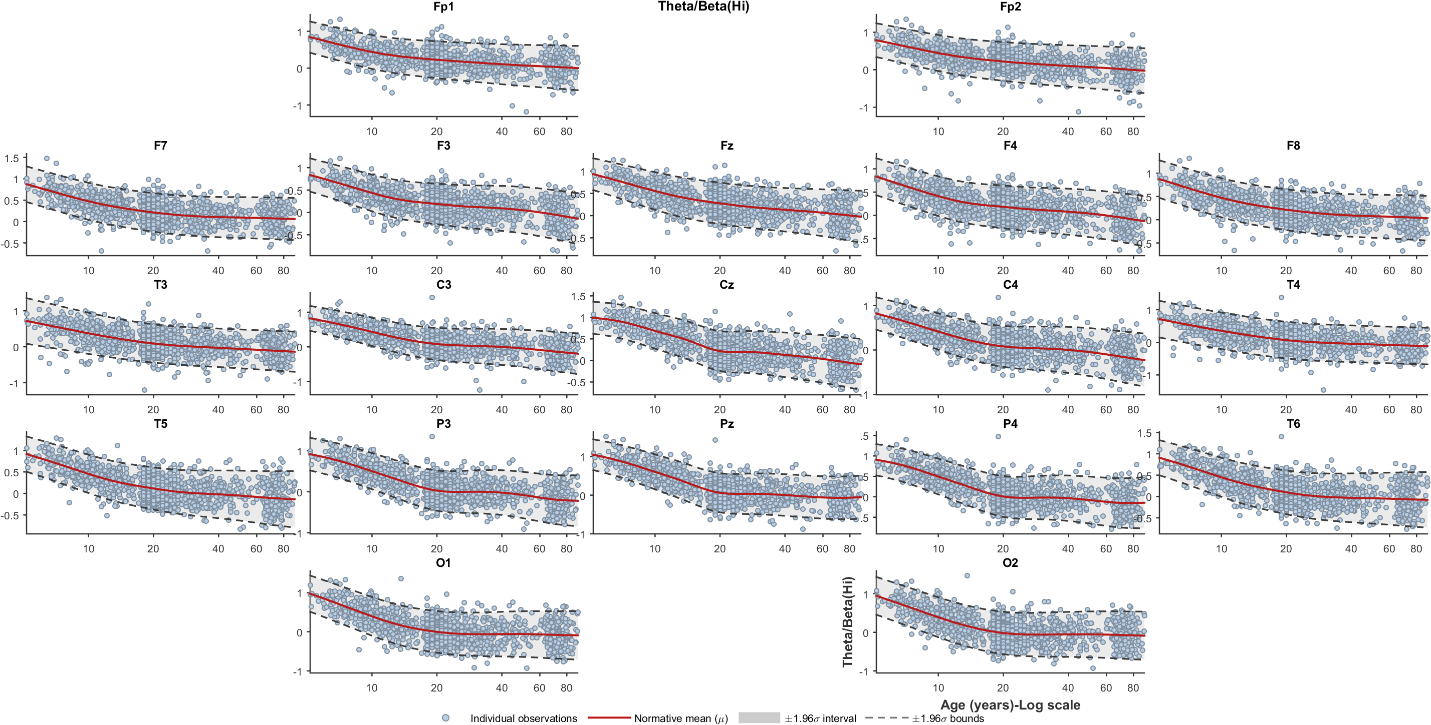


**Sup. Figure 5.** Theta/High-Beta Ratio, defined as ln[θ(ch)] − ln[β_Hi(ch)], where β_Hi denotes power in the high-beta sub-band (~15–30 Hz). The developmental trajectory closely parallels that of the broadband TBR (Supplementary Figure 1), with a pronounced age-dependent decrease from childhood to early adulthood across all electrodes. Values are systematically higher than broadband TBR at comparable ages, reflecting the lower absolute power in the high-beta sub-band relative to the full beta band. The frontal predominance of the ratio and its sensitivity to attentional regulation are preserved, making this index a complementary marker of cortical arousal with particular relevance for neurofeedback protocols targeting the high-beta range (Arns et al. 2013; Ogrim et al. 2012).


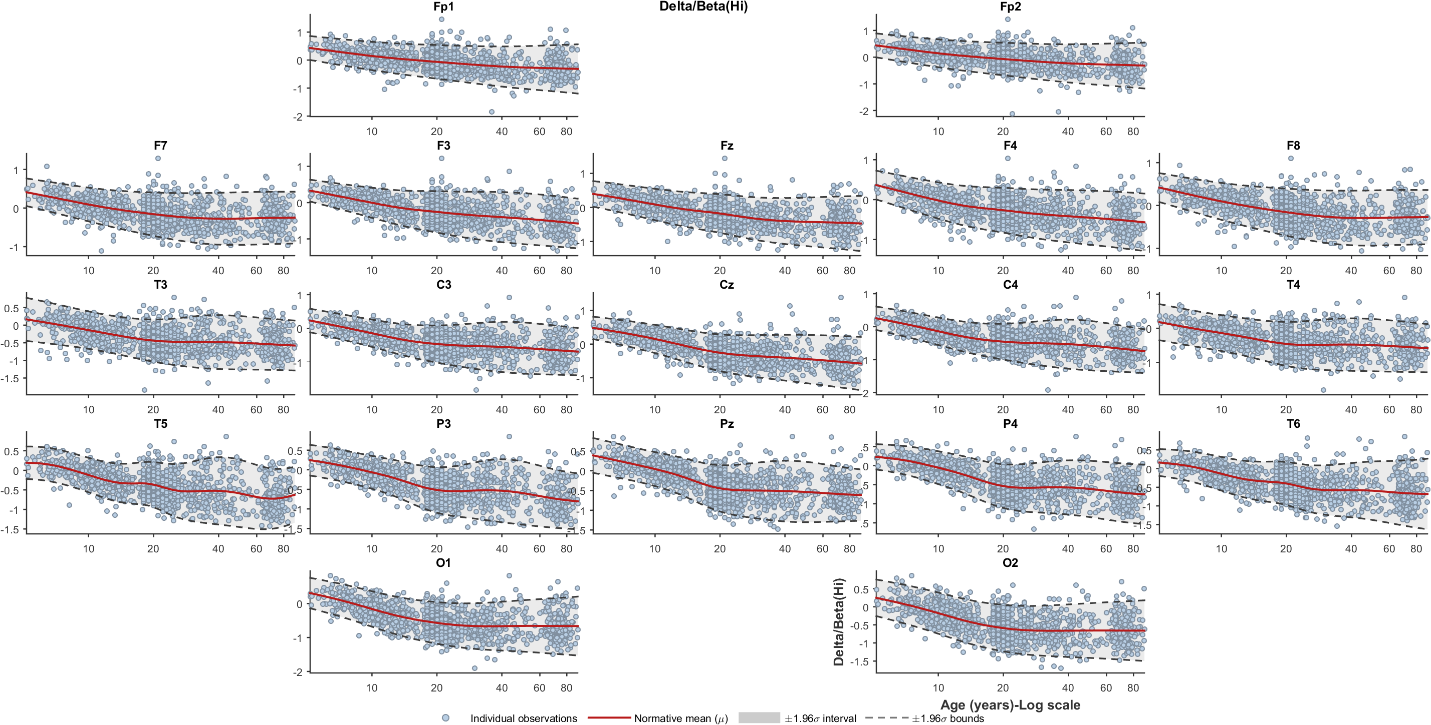


**Sup. Figure 6**. Delta/High-Beta Ratio, defined as ln[δ(ch)] − ln[β_Hi(ch)], where β_Hi denotes power in the high-beta sub-band (~15–30 Hz). The index captures the balance between infra-slow delta oscillations and fast cortical activity, and shows a pronounced age-dependent decrease across all electrodes, most marked in frontal and central regions. The steeper descent compared to TBR reflects the combined contribution of both delta reduction and high-beta maturation during development. Notably, values at temporal electrodes (T3, T4, T5, T6) show greater inter-individual variability and a wider normative band relative to midline sites, consistent with the higher variability of delta activity in lateral temporal regions. Elevated Delta/Beta(Hi) has been associated with cortical hypoactivation and is sensitive to disorders of consciousness and severe attentional dysregulation.


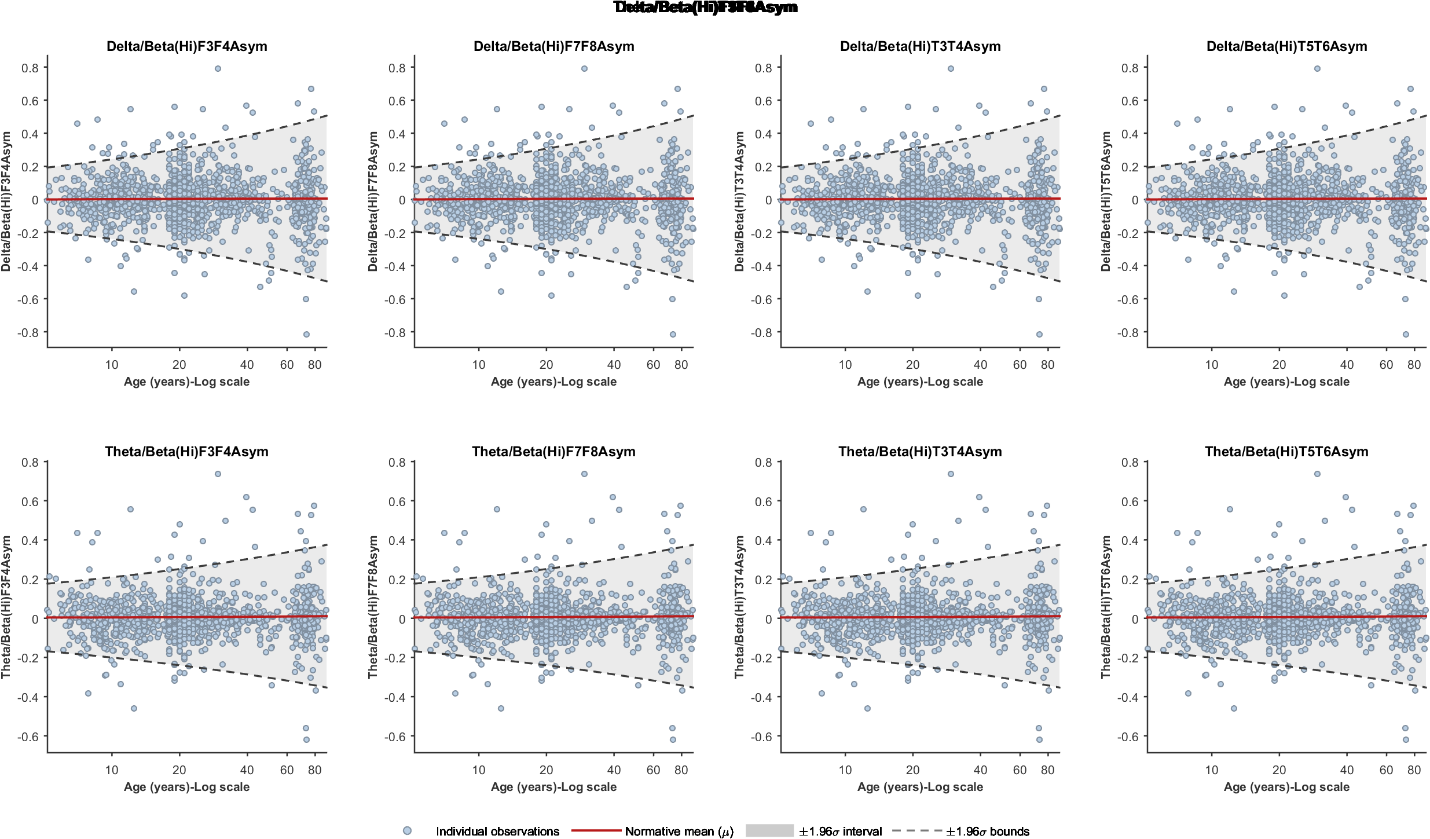


**Sup. Figure 7. Age-dependent normative centile curves for hemispheric ratio asymmetry indices based on Delta/Beta(Hi) and Theta/Beta(Hi) at four homologous electrode pairs.** Each panel displays the normative trajectory for one asymmetry index at one electrode pair.

Top row — Delta/Beta(Hi) asymmetry: computed at four homologous pairs: F3–F4 (dorsolateral prefrontal), F7–F8 (lateral prefrontal), T3–T4 (temporal), and T5–T6 (posterior temporal). Bottom row — Theta/Beta(Hi) asymmetry: computed at the same four pairs.

A normative mean near zero reflects hemispheric symmetry, consistent with the healthy population. The ±1.96σ normative band widens with age, particularly at frontal pairs, reflecting increased inter-individual variability in hemispheric lateralization in adulthood and older age. Delta/Beta(Hi) asymmetry indices show a modest age-dependent decrease in normative dispersion at temporal sites, whereas Theta/Beta(Hi) asymmetry at F3–F4 shows a slight increase in the normative band width with age, consistent with the known developmental increase in frontal alpha asymmetry variability. Positive values indicate left-hemisphere dominance of the respective ratio; negative values indicate right-hemisphere dominance.


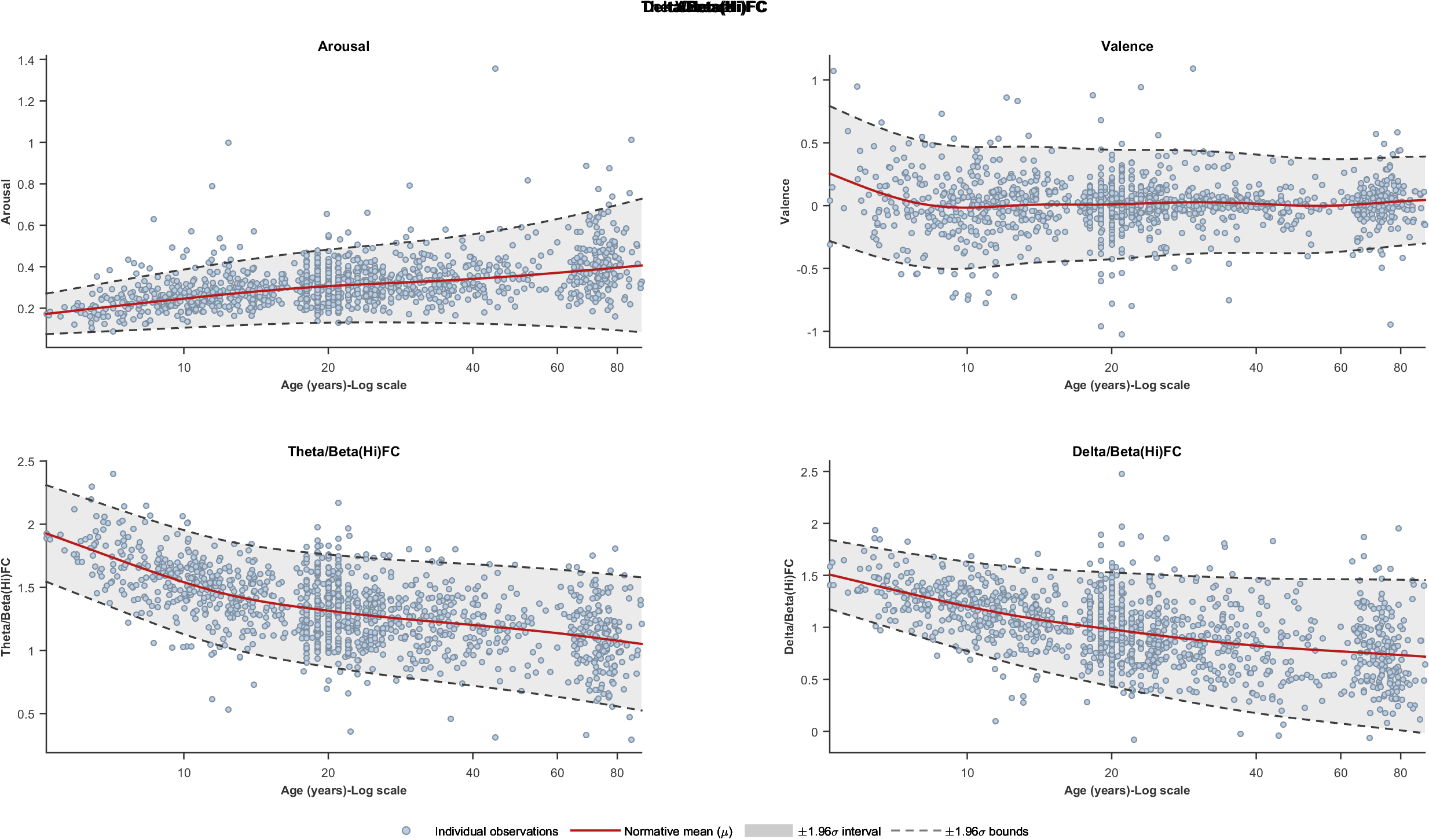


**Sup. Figure 8. Age-dependent normative centile curves for global functional state indices and frontocentral spectral ratios.** **(Top left) Arousal Index (AI):** defined as β̄_F / (δ̄_F + θ̄_F), where the overbar denotes the mean over the frontal cluster F3, F4. The normative trajectory increases monotonically with age, reflecting progressive frontal fast-wave dominance across the lifespan, with the largest inter-individual variability in childhood and early adolescence.

**(Top right) Valence Index:** defined as α(F3)/β(F3) − α(F4)/β(F4). The normative mean remains near zero across the lifespan, consistent with hemispheric symmetry in the healthy population. The normative band is wide and nearly constant across ages, reflecting high inter-individual variability in frontal lateralization that is independent of age — a well-known property of this index in healthy samples (Allen et al. 2018; Davidson 1998). The vertical clustering of observations around ages 15–20 reflects the higher density of adolescent subjects in the normative database.

**(Bottom left) FrontoCentral Theta/Beta(Hi) [FC-TBR(Hi)]:** defined as ln[θ̄_{F3,F4,Cz}] − ln[β_Hi̅_{F3,F4,Cz}]. The trajectory shows a pronounced age-dependent decrease from childhood to adulthood, with a wide normative band in childhood that narrows progressively, consistent with the maturation of frontocentral attentional regulation networks.

**(Bottom right) FrontoCentral Delta/Beta(Hi) [FC-DBR(Hi)]:** defined as ln[δ̄_{F3,F4,Cz}] − ln[β_Hi̅_{F3,F4,Cz}]. The developmental pattern parallels FC-TBR(Hi) but shows a steeper descent and wider normative band, particularly in childhood, reflecting the additional contribution of delta reduction to frontocentral maturational change.


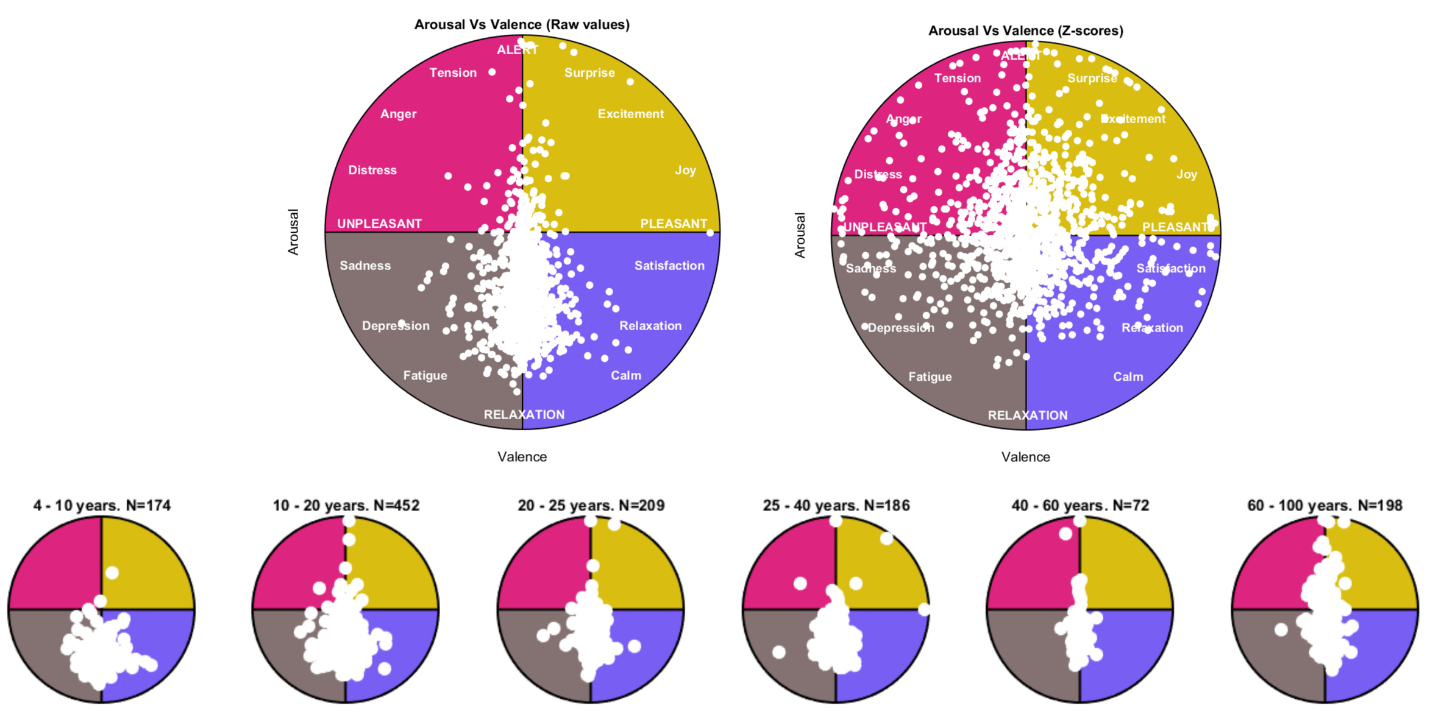


**Sup. Figure 9. Arousal–Valence distribution of the normative sample mapped onto the circumplex model of affect.** Each white dot represents one individual. The circular space is partitioned into four quadrants according to the two-dimensional valence–arousal framework (Russell, 1980): high arousal / pleasant (yellow, upper right), high arousal / unpleasant (pink, upper left), low arousal / pleasant (blue, lower right), and low arousal / unpleasant (grey, lower left). Emotional states labelled within each quadrant correspond to prototypical affect categories. **Top row:** full-sample distributions plotted using raw index values (left) and age-corrected Z-scores (right; N = 1,564). The shift toward the center observed in the Z-score panel reflects the removal of age-related variance in both indices. **Bottom row:** raw-value distributions stratified by age group (4–10 yr, N = 174; 10–20 yr, N = 452; 20–25 yr, N = 209; 25–40 yr, N = 186; 40–60 yr, N = 72; 60–100 yr, N = 198), illustrating the developmental trajectory of resting-state affective tone. Younger participants cluster preferentially in the low-arousal quadrants, whereas the distribution broadens and shifts toward higher arousal in adolescence and early adulthood.

**
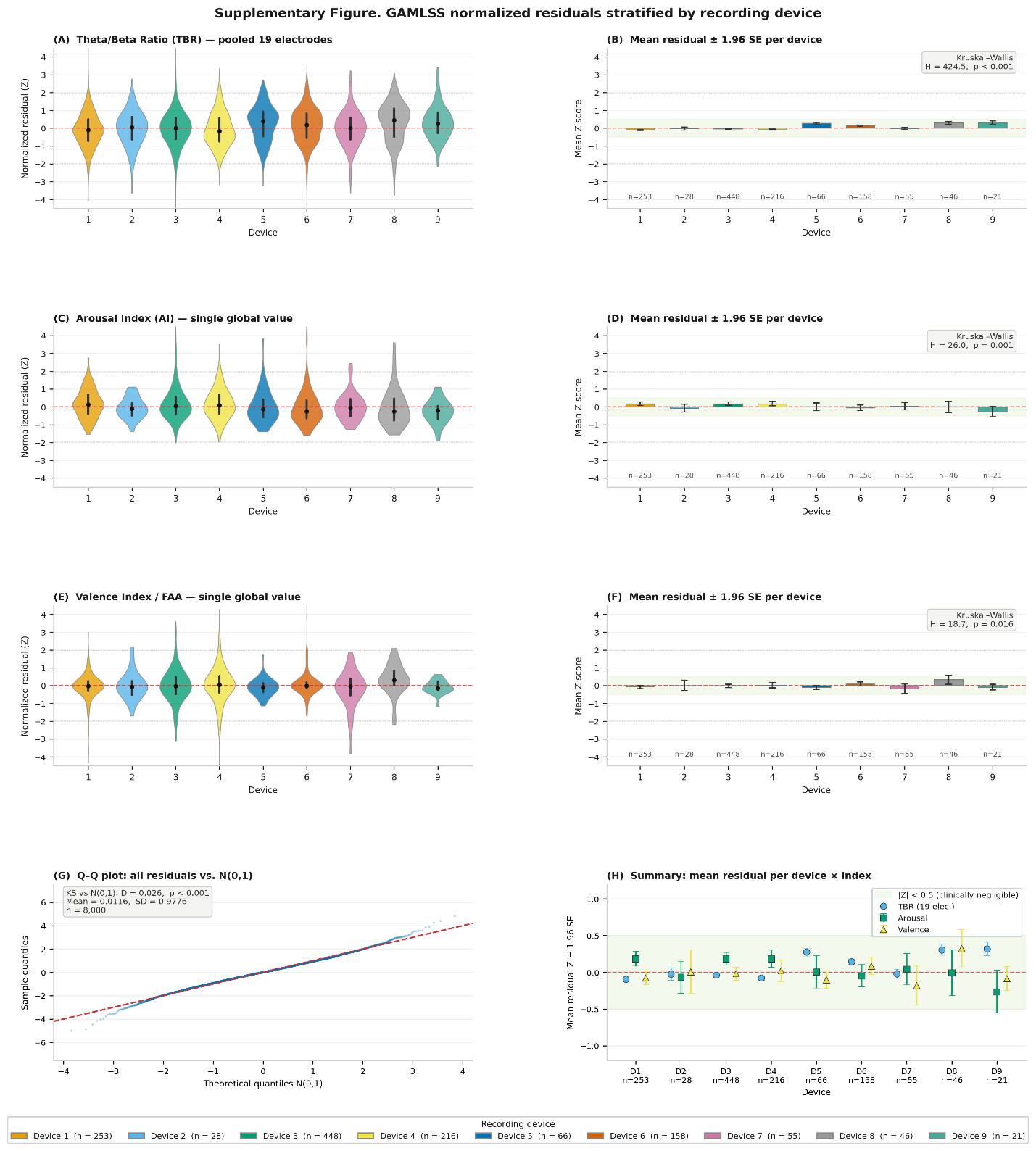
**

**Sup. Figure 10**: **GAMLSS normalized residuals stratified by recording device.** Normalized quantile residuals from GAMLSS models for three representative indices (TBR, Arousal, Valence; n = 1,564; 9 recording devices). Panels A, C, E: per-device residual distributions (violin plots); black dot = median, bar = IQR; dashed red line = Z = 0; dotted lines = ±1.96. Panels B, D, F: mean residual ± 1.96 SE per device; green band indicates the |Z| < 0.5 region. Panel G: Q–Q plot of all pooled residuals against the standard normal (n = 8,000 random subsample). Panel H: cross-index summary of mean device residuals. Although Kruskal–Wallis tests detect statistically significant inter-device differences (driven by the large sample size), all mean deviations remain within |Z| < 0.5 — well below any clinically meaningful threshold — confirming that ESN normalization adequately removes equipment-related amplitude variability before normative modeling. The overall residual distribution is approximately normal (Mean ≈ 0, SD ≈ 1.0), consistent with a well-calibrated normative model.

**
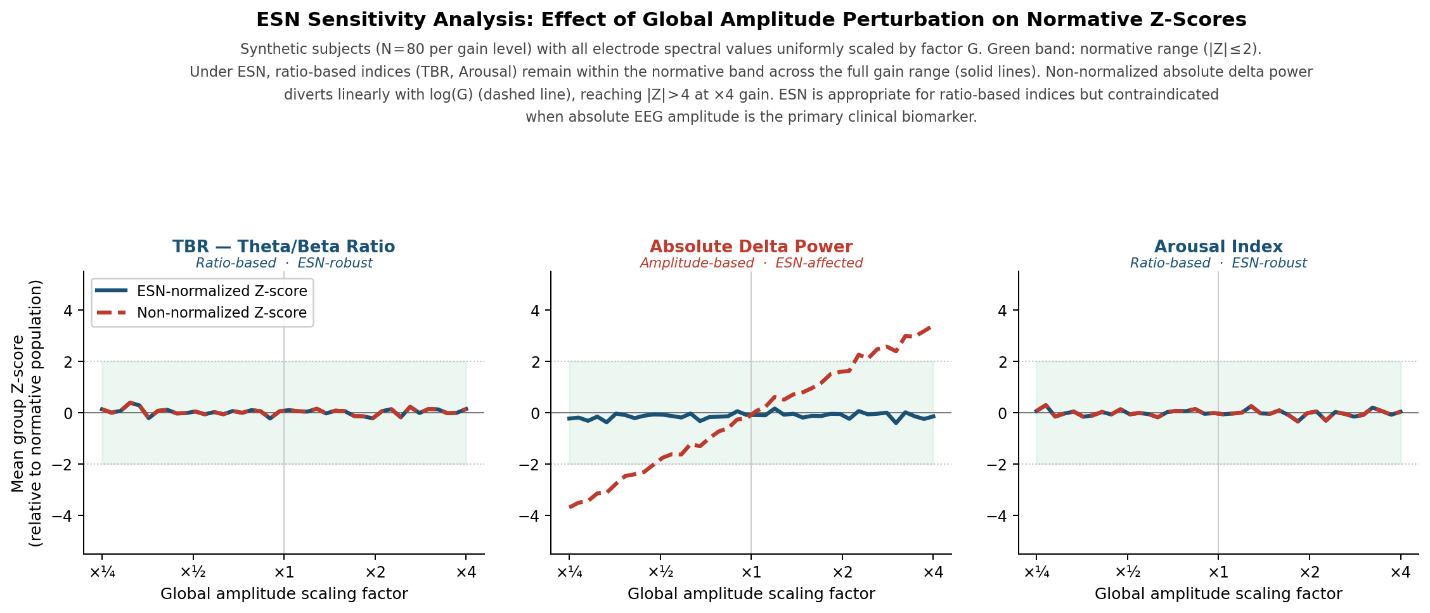
**

**Supplementary Figure 11. S_ESN. Proof-of-concept sensitivity analysis of Electrode-level Spectral Normalization (ESN) against global EEG amplitude perturbation.**

Synthetic normative subjects (N = 300) were generated with realistic log-normal spectral power profiles across four canonical frequency bands at global and frontal electrode clusters. Test subjects (N = 80 per gain level) were constructed by applying a uniform multiplicative gain factor G — ranging from ×¼ to ×4 on a logarithmic scale — to all electrode spectral values simultaneously, simulating the kind of global amplitude differences that arise from inter-individual variability in skull conductivity, electrode impedance, or between-device gain differences. For each gain level, the mean group Z-score was computed for three representative indices relative to the normative population distribution, under two conditions: with ESN normalization applied (solid navy line) and without (dashed red line). The three indices represent distinct index families: (A) TBR — Theta/Beta Ratio (ratio-based, computed within the same electrode); (B) Absolute Delta Power (amplitude-based, log-transformed); (C) Arousal Index (ratio-based, computed across frontal band powers). Green shading indicates the normative range (|Z| ≤ 2); dotted horizontal lines mark ±2 SD; the vertical line at ×1 indicates no perturbation.

Under ESN, ratio-based indices (panels A and C) produce Z-scores that remain within the normative band across the entire gain range, demonstrating that ESN effectively removes global multiplicative amplitude differences for indices whose computation is invariant to a common scaling factor. In contrast, non-normalized absolute delta power (panel B, dashed line) diverges proportionally to log(G), reaching |Z| > 4 at ×4 gain and |Z| < −4 at ×¼ gain — confirming that ESN masks the very signal that would be clinically informative when absolute EEG amplitude is the primary biomarker. Notably, even the ESN-normalized absolute delta power (panel B, solid line) remains near zero, illustrating that ESN renders absolute amplitude indices uninformative. This simulation provides the theoretical and empirical basis for the contraindication stated in Section 2.4.3: ESN should not be applied when the clinical question concerns globally elevated or suppressed EEG amplitude (e.g., diffuse encephalopathy, burst suppression).

**
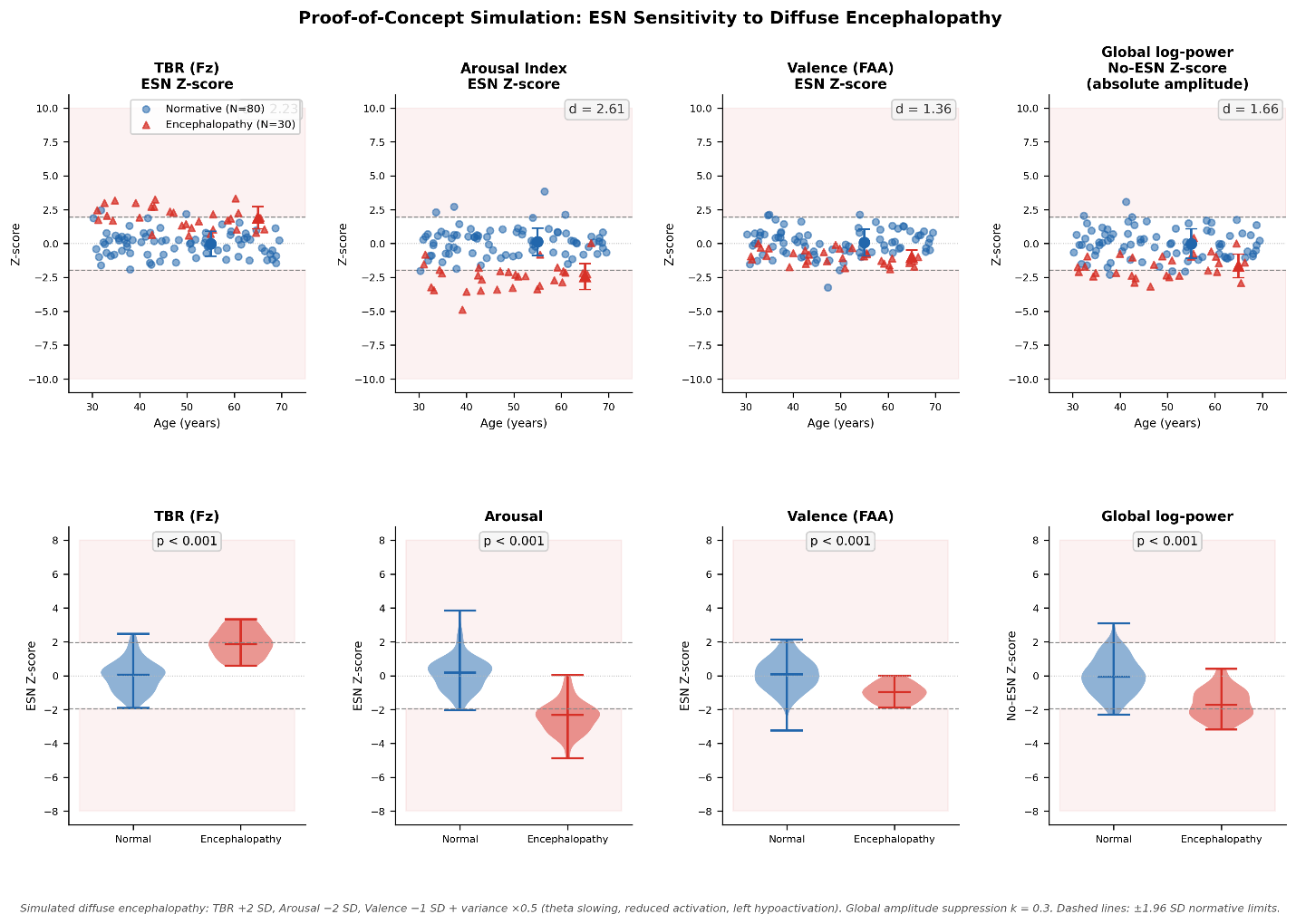
**

**Supplementary Figure 12. Proof-of-concept simulation illustrating ESN sensitivity to diffuse encephalopathy.** Synthetic normative subjects (N = 80, blue circles) were generated by sampling from the age-dependent GAMLSS normative distributions fitted in this study (ages 30–70 yr). Simulated diffuse encephalopathy subjects (N = 30, red triangles) were generated with systematically altered spectral profiles: TBR +0.55 log-ratio units (+2 SD), Arousal −0.30 log-ratio units (−2 SD), and Valence −0.19 log-ratio units (−1 SD) with variance reduced to 50% of normative. An additional global amplitude suppression of k = 0.3 (ln k ≈ −1.20) was applied to simulate generalized voltage attenuation. **Top row:** ESN-normalized Z-scores as a function of age for TBR, Arousal, and Valence (panels 1–3), and global log-power Z-score without ESN normalization (panel 4). Cohen's d effect sizes are shown for each index. Dashed lines indicate ±1.96 SD normative limits. **Bottom row:** Violin plots of Z-score distributions for normative vs. encephalopathy groups with Mann-Whitney U test p-values. ESN-normalized ratio indices detect the pathological spectral pattern across all three indices (TBR: d = 2.23; Arousal: d = 2.61; Valence: d = 1.36; all p < 0.001) despite global amplitude suppression, confirming that ratio-based ESN-normalized Z-scores are sensitive to encephalopathic spectral reorganization. Global log-power without ESN detects amplitude suppression (d = 1.66, p < 0.001) but not spectral reorganization, illustrating the complementary nature of amplitude-based and ratio-based normative approaches and delineating the scenarios where ESN normalization is appropriate versus contraindicated.

**
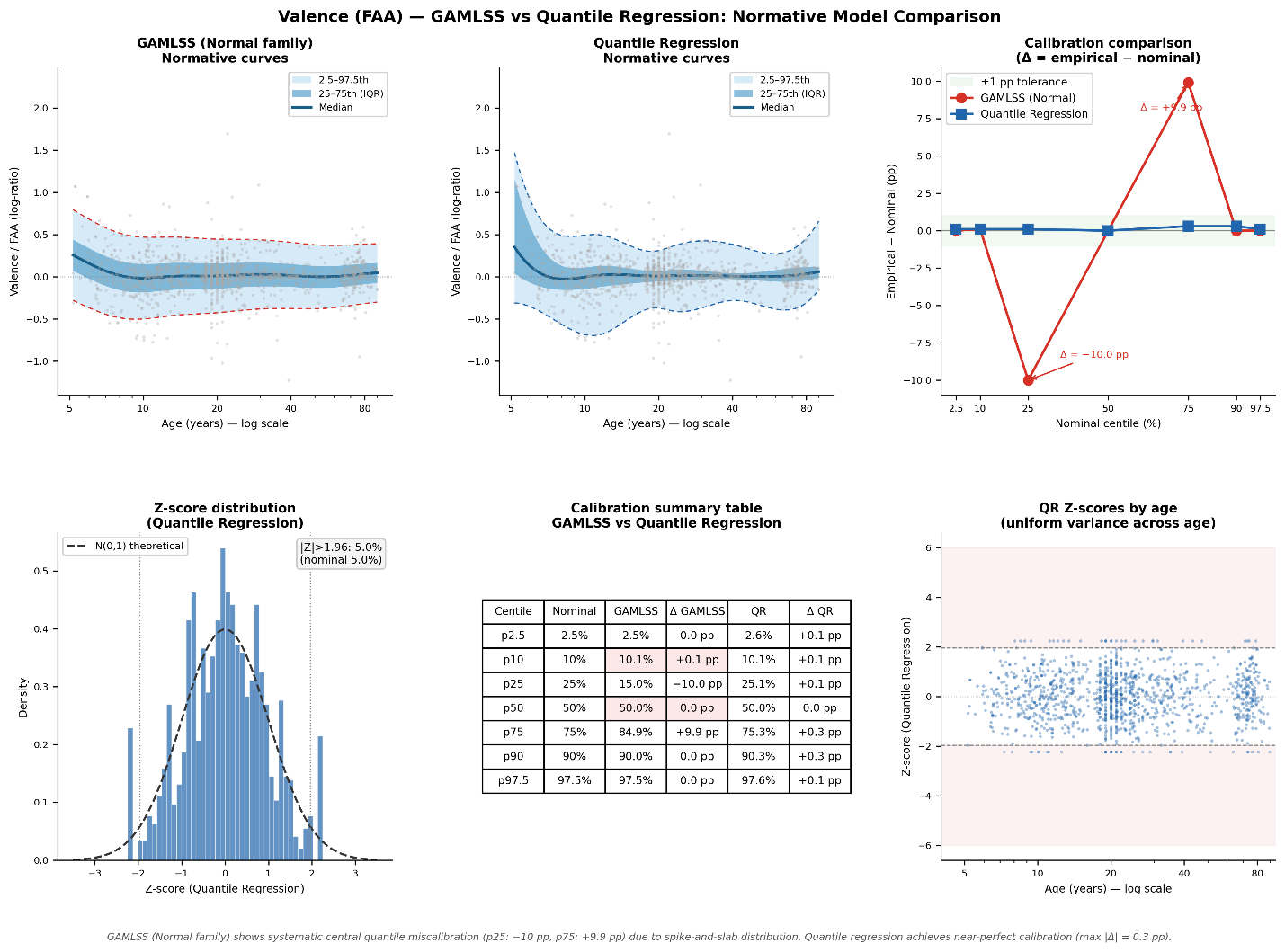
**

***Supplementary Figure 13. Comparison of GAMLSS (Normal family) and quantile regression normative models for the Valence index.***

*Top row. (A) Age-dependent normative curves fitted with GAMLSS (Normal family). Shaded bands indicate the 2.5–97.5th (light blue) and 25–75th IQR (dark blue) centile intervals; dashed red lines mark the 2.5th and 97.5th centiles; the solid dark line is the median (50th centile). Individual observations are shown as grey dots. The IQR band is visibly over-wide relative to the data density, reflecting the systematic over-dispersion of the Normal family for this index. (B) Age-dependent normative curves fitted with quantile regression (B-spline, cubic, 4 internal knots, log-age scale). The IQR band closely tracks the empirical data density across all ages. (C) Calibration comparison: difference between empirical and nominal centile coverage (Δ = empirical − nominal, in percentage points) for GAMLSS (red circles) and quantile regression (blue squares). The green band indicates ±1 pp tolerance. GAMLSS shows systematic miscalibration at p25 (Δ = −10.0 pp) and p75 (Δ = +9.9 pp), consistent with over-estimation of dispersion in the central range. Quantile regression achieves near-perfect calibration at all centiles (max |Δ| = 0.3 pp).*

*Bottom row. (D) Distribution of quantile regression Z-scores (Φ⁻¹ of empirical percentile) overlaid with the theoretical N(0,1) density (dashed line). The proportion outside ±1.96 SD is 4.96%, close to the nominal 5.0%, confirming global calibration. (E) Summary calibration table comparing empirical centile coverage for GAMLSS and quantile regression. Cells highlighted in red indicate centiles where GAMLSS miscalibration exceeds 5 pp. (F) Quantile regression Z-scores as a function of age (log scale), showing uniform variance across the full age range with no systematic age-dependent bias.*

*The quantile regression model is recommended as the primary normative reference for the Valence index. GAMLSS-based Z-scores for this index should be restricted to extreme deviations (|Z| > 2), where both models converge and parametric miscalibration in the central range does not affect clinical interpretation.*

**
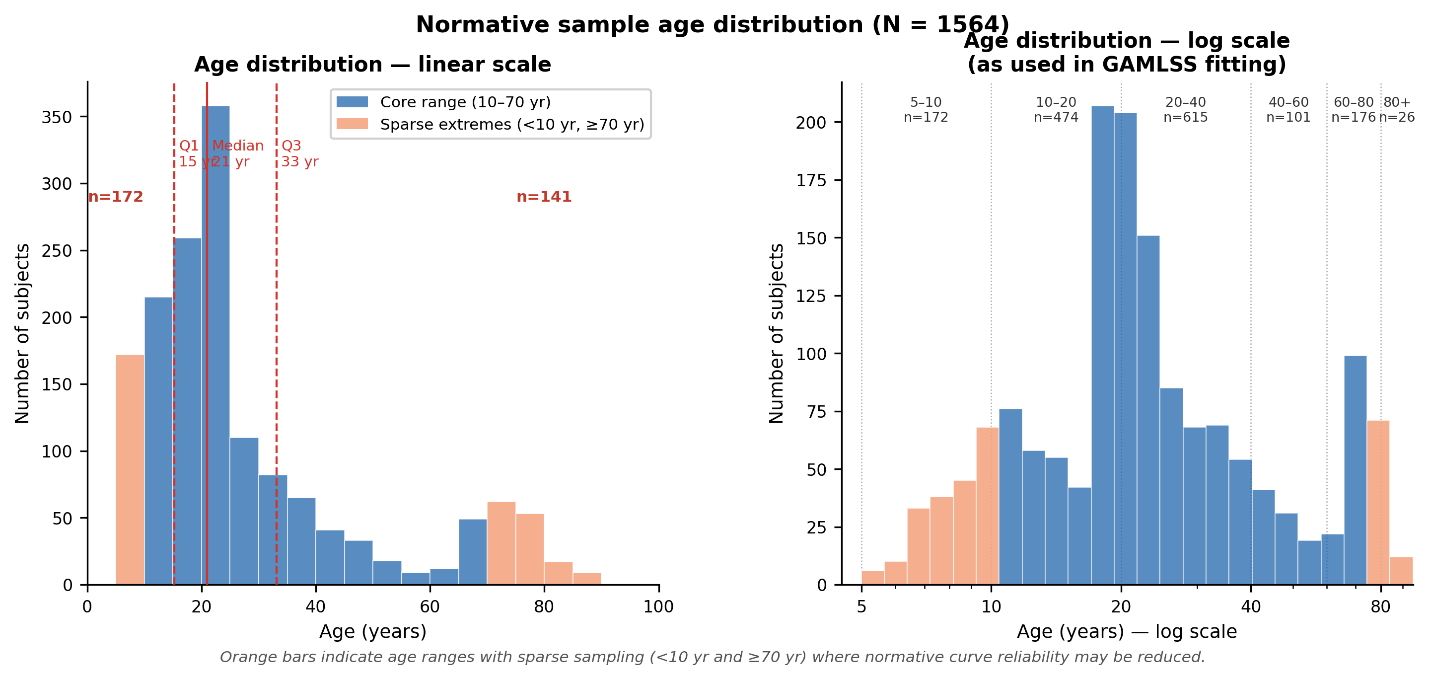
Supplementary Figure 14. Age distribution of the normative sample (N = 1,564).** **(A)** Histogram with 5-year bins on a linear age scale. Vertical lines indicate the 25th percentile (15 yr), median (21 yr), and 75th percentile (33 yr). **(B)** Histogram on a logarithmic age scale, matching the scale used in GAMLSS model fitting, with subjects stratified by age group. Orange bars indicate age ranges with sparse sampling (< 10 yr, n = 172; ≥ 70 yr, n = 141) where normative curve reliability may be reduced relative to the core 10–70 yr range (n = 1,251). Users applying these norms to individuals outside the 10–70 yr range should interpret Z-scores with additional caution.

**Supplementary Table 1. Empirical calibration of GAMLSS normative models**

*Percentage of normative sample with normalized quantile residuals |Z| > 1.96, evaluated per index using R's internal residual diagnostics. Under a perfectly calibrated model, the expected rate is 5.0%.*

| **Index** | **n** | **Scope** | **Family** | **Transform** | **% \|Z\| > 1.96** | **Δ from 5%** |
| --- | --- | --- | --- | --- | --- | --- |
| TBR | 1,564 | 19 electrodes (pooled) | NO | — | 4.95% (range: 4.0–5.7%) | -0.05 pp |
| Arousal Index | 1,564 | Global | BCT | log† | 5.03% | +0.03 pp |
| Valence | 1,564 | Global | NO | — | 5.11% | +0.11 pp |

pp = percentage points. NO = Normal; BCT = Box-Cox t distribution. † Transform was specified, but BCT was fitted on raw data (values > 0); the ν parameter of BCT absorbs asymmetry, making the log pre-transform redundant. TBR range reflects variability across the 19 individual electrode models. All three indices show adequate calibration, with deviations from the nominal 5% rate below 0.2 pp.

**Supplementary Table 2. Empirical centile coverage of GAMLSS normative models**

*Empirical percentage of the normative sample falling below each centile threshold, derived by comparing each subject's observed index value against the age-interpolated centile curve. Values in parentheses indicate the deviation (Δ) from the nominal level. Under perfect calibration, all deviations are equal to 0.*

| **Index** | **Scope** | **Family** | **Transf** | **p2.5** | **p10** | **p25** | **p50** | **p75** | **p90** | **p97.5** |
| --- | --- | --- | --- | --- | --- | --- | --- | --- | --- | --- |
|  |  |  |  | ***(exp. 2.5%)*** | ***(exp. 10%)*** | ***(exp. 25%)*** | ***(exp. 50%)*** | ***(exp. 75%)*** | ***(exp. 90%)*** | ***(exp. 97.5%)*** |
| ***TBR*** | ***19 elec.*** | ***NO*** | ***—*** | ***2.5 (-0.0)*** | ***9.2 (-0.8)*** | ***23.7 (-1.3)*** | ***49.9 (-0.1)*** | ***75.7 (+0.7)*** | ***91.0 (+1.0)*** | ***97.5 (+0.0)*** |
| ***Arousal*** | ***global*** | ***BCT*** | ***log†*** | ***2.6 (+0.1)*** | ***10.4 (+0.4)*** | ***25.9 (+0.9)*** | ***49.0 (-1.0)*** | ***74.8 (-0.2)*** | ***90.3 (+0.3)*** | ***97.6 (+0.1)*** |
| ***Valence*** | ***global*** | ***NO*** | ***—*** | ***3.1 (+0.6)*** | ***6.7 (-3.3) ‡*** | ***15.0 (-10.0) ‡*** | ***50.3 (+0.3)*** | ***84.9 (+9.9) ‡*** | ***93.6 (+3.6) ‡*** | ***97.9 (+0.4)*** |

*pp = percentage points. NO = Normal; BCT = Box-Cox t distribution. TBR was pooled across 19 electrode models. ‡ |Δ| > 3 pp. † BCT fitted on raw data; log-transform redundant (absorbed by BCT's ν parameter).*

*TBR and Arousal show excellent calibration across all centiles (max |Δ| < 1.4 pp and < 1.0 pp, respectively). Valence (NO) shows systematic underdispersion in the central quantiles (p25: -10.0 pp; p75: +9.9 pp), consistent with leptokurtic residuals not captured by the Normal family; a BCT refit is recommended for future versions.*

***Supplementary Table 3. Adaptive distributional family selection criteria in GAMLSS normative modeling***

| **Condition** | **Criteria met (out of 3)** | **Family sequence** | **Rationale** |
| --- | --- | --- | --- |
| Raw data strictly positive (y > 0) | ≥ 2 | BCT → BCPE → NO | BCT handles asymmetry and heavy tails via shape parameters ν and τ; log pre-transform is redundant |
| Raw data includes negative values | ≥ 2 | NO directly | Empirically verified: JSU/SHASHo overestimate σ for spike-and-slab distributions (e.g. the Valence index) |
| Raw data strictly positive (y > 0) | 1 | BCPE → NO | Moderate departure from normality; conservative family |
| Any sign | 0 | NO + requested transform | Near-normal distribution; apply log/sqrt pre-transform if specified |

*Three diagnostic criteria evaluated on raw (pre-transformation) data: (1) excess kurtosis > 1 (i.e. kurtosis > 4); (2) |skewness| > 0.5; (3) Shapiro–Wilk test p < 0.05 on a subsample of up to 2,000 observations. BCT = Box-Cox t; BCPE = Box-Cox Power Exponential; NO = Normal. Arrows indicate fallback order if the primary family fails to converge. The criterion is evaluated on raw data regardless of any pre-transformation specified in gamlss_meta.csv*, because BCT and BCPE require y > 0, which holds in the original scale for positive indices such as the Arousal Index.**

***Supplementary Table 4. Kruskal-Wallis test for inter-device differences***

| **Index** | **N** | **H (df=8)** | **p** | **η²** | **max\|Z̄\|** |
| --- | --- | --- | --- | --- | --- |
| TBR | 1,291 | 26.65 | 0.0008 | 0.015 | 0.32 |
| TAR | 1,291 | 40.93 | <0.001 | 0.026 | 0.75 |
| ABR | 1,291 | 47.07 | <0.001 | 0.031 | 0.41 |
| Arousal | 1,291 | 23.02 | 0.003 | 0.012 | 0.44 |
| Valence | 1,291 | 18.65 | 0.017 | 0.008 | 0.33 |
| CognAf | 1,291 | 17.85 | 0.022 | 0.008 | 0.34 |

*Kruskal-Wallis test for inter-device differences in mean GAMLSS normalized residuals across 9 recording devices (df = 8 for all indices). η² = effect size (epsilon-squared). max|Z̄| = maximum absolute mean residual across devices. All effect sizes are negligible (η² < 0.05) and all device means fall within |Z| < 0.75, well below any clinically meaningful threshold.*
